# Association of Patient Outcomes With Bundled Payments Among Hospitalized Patients Attributed to Accountable Care Organizations

**DOI:** 10.1001/jamahealthforum.2021.2131

**Published:** 2021-08-20

**Authors:** Amol S. Navathe, Joshua M. Liao, Erkuan Wang, Ulysses Isidro, Jingsan Zhu, Deborah S. Cousins, Rachel M. Werner

**Affiliations:** 1 Corporal Michael J. Crescenz VA Medical Center, Philadelphia, Pennsylvania; 2 Leonard Davis Institute of Health Economics, University of Pennsylvania, Philadelphia; 3 Department of Medical Ethics and Health Policy, Perelman School of Medicine, University of Pennsylvania, Philadelphia; 4 Department of Medicine, University of Washington School of Medicine, Seattle; 5 Department of Medicine, Perelman School of Medicine, University of Pennsylvania, Philadelphia

## Abstract

**Importance:**

It is unknown how outcomes are affected when patients receive care under bundled payment and accountable care organization (ACO) programs simultaneously.

**Objective:**

To evaluate whether outcomes in the Medicare Bundled Payments for Care Improvement (BPCI) program differed depending on whether patients were attributed to ACOs in the Medicare Shared Savings Program.

**Design, Setting, and Participants:**

This cohort study was conducted using Medicare claims data from January 1, 2011, to September 30, 2016, and difference-in-differences analysis to compare episode outcomes for patients admitted to BPCI vs non-BPCI hospitals. Outcomes were stratified for patients who were and were not attributed to an ACO. Participants included Medicare fee-for-service beneficiaries receiving care for medical and surgical episodes at US hospitals. Data were analyzed between October 1, 2018, and June 10, 2021.

**Exposures:**

Hospitalization for any of the 48 episodes (24 medical, 24 surgical) included in the BPCI at US hospitals participating in the BPCI for those episodes.

**Main Outcomes and Measures:**

The primary outcome was change in 90-day postdischarge institutional spending, and secondary outcomes included changes in quality and utilization.

**Results:**

A total of 7 108 146 beneficiaries (mean [SD] age, 76.9 [12.2] years; 4 101 081 women [58%]) received care for medical episodes, and 3 675 962 beneficiaries (mean [SD] age, 74.8 [10.1] years; 2 074 921 women [56%]) received care for surgical episodes. Compared with patients who were not attributed to ACOs, the association between bundled payments and changes in postdischarge institutional spending was larger among patients attributed to ACOs (–$323 difference; 95% CI, –$607 to –$39; *P* = .03) for medical episodes, but not surgical episodes. Attribution to an ACO also increased the strength of the association between bundled payments and changes in 90-day readmissions for both medical episodes (−0.98 percentage point difference; 95% CI, –1.55 to –0.41; *P* = .001) and surgical episodes (−0.84 percentage point difference; 95% CI, −1.32 to −0.35; *P* = .001).

**Conclusions and Relevance:**

In this cohort study, compared with inclusion in bundled payments alone, simultaneous inclusion in both ACOs and bundled payment programs was associated with lower institutional postacute care spending and readmissions for medical episodes and lower readmissions but not spending for surgical episodes. Receiving care under models such as ACOs may improve episode outcomes under bundled payments.

## Key Points

**Question:**

Is receiving care simultaneously under a Medicare accountable care organization (ACO) and bundled payments associated with better patient outcomes compared with bundled payments alone?

**Findings:**

In this cohort study of 9 850 080 Medicare beneficiaries, simultaneous inclusion in both ACOs and bundled payments was associated with lower spending on institutional postacute care, fewer readmissions for medical episodes, and fewer readmissions only for surgical episodes compared with inclusion in bundled payments alone.

**Meaning:**

These findings suggest that receiving care under models such as ACOs may improve patient outcomes under bundled payments.

## Teaser

This cohort study evaluates whether simultaneous inclusion in a Medicare accountable care organization and a bundled payment program was associated with better patient outcomes, compared with bundled payments alone.

## Introduction

Participation in value-based alternative payment models continues to grow nationwide.^1^ More than one-third of all health care payments in the US are now tied to these models, driven largely by policies implemented in the Medicare fee-for-service program by the Centers for Medicare and Medicaid Services (CMS). This trend is likely to continue as the agency reinforces its goal of shifting to value-based payments.^2,3,4,5^

Growing participation in alternative payment models has given rise to situations in which beneficiaries receive care simultaneously under multiple models. This issue has created significant concerns for policy makers.^6,7,8^ For example, the CMS cited “model overlap issues that impact providers already participating in [alternative payment models]” in the rationale to cancel the Episode Payment Model, a bundled payment program for cardiac conditions.^6^^(p57069)^

These dynamics are particularly salient for Accountable Care Organizations (ACOs) and bundled payments, 2 of the CMS’ most prominent alternative payment models. Patient inclusion in both has increased over time, with approximately 30% of all patients who receive care under bundled payments also being attributed to an ACO.^9^ The 2 models could have additive benefits, as bundles have focused on utilization in hospital and postacute settings, and ACOs may contribute additional benefits in those settings through their focus on utilization across the care continuum.^10,11,12,13,14,15,16,17,18,19,20,21,22,23,24,25,26,27^ Conversely, the payment models may produce some or no additive benefits if simultaneous care under both involves duplicative or uncoordinated care.

However, despite the continued commitment of the CMS to scaling up both ACOs and bundled payments, current policy financially penalizes ACOs when attributed beneficiaries receive care from bundled payment participants—a dynamic that may dissuade patient inclusion in both payment models and fail to capture any potential additive benefits.^28^ These issues are particularly important for Medicare policy makers, whose statutory authority to expand alternative payment models is based only on single-model evaluations, not evaluations that account for patient inclusion in multiple models. Yet, it is unknown whether and to what extent there are any additive benefits from patients’ inclusion under ACOs on episode outcomes under bundled payments.

To address this gap, we evaluated the association of episode outcomes with Medicare’s largest completed bundled payment model, the Bundled Payments for Care Improvement (BPCI) initiative, among hospitalized patients attributed to Medicare’s largest ACO model, the Medicare Shared Savings Program (MSSP).^29,30^

## Methods

This cohort study included data for 9 850 080 Medicare beneficiaries from January 1, 2011, to September 30, 2016, and a difference-in-differences analysis was performed to compare episode outcomes for patients admitted to BPCI vs non-BPCI hospitals. The study was approved by the University of Pennsylvania’s institutional review board, and a waiver of informed consent was provided, owing to a lack of feasibility given the large size of the retrospective cohort. We followed the Strengthening the Reporting of Observational Studies in Epidemiology (STROBE) reporting guideline.^31^

### Rationale for Additive Effects Between Bundled Payments and ACOs

Both payment models (bundled payments and ACOs) were designed to improve coordination between care settings, with bundled payments more targeted on coordination between hospital and postacute settings and ACOs more broadly focused on coordination across the care continuum between outpatient, hospital, and postacute settings. The mechanisms through which they have achieved savings are thus, in part, complementary. Accountable care organizations have been particularly effective in reducing avoidable hospitalizations, whereas bundles have been effective in reducing expensive care during and after hospitalization.^10,11,12,13,14,15,16,17,18,19,20,21,22,23,24,25,26,27^ As such, there is the possibility that the 2 models complement each other in ways that could produce additive benefits. For instance, bundled payments may target transitions between hospitals and postacute care facilities, whereas ACOs target transitions from postacute facilities back into ongoing outpatient care. Conversely, because they both encompass the period spanning hospitalization and discharge to postacute care, the 2 payment models could lead to duplicative services that substitute for other services and do not achieve additive benefits. For example, both a hospital in bundled payments and an ACO could set up separate preferred networks of skilled nursing facilities. Care redesign under bundled payments and ACOs may be partial complements or substitutes to one another, creating partial rather than fully additive benefits.

### Study Sample and Study Period

We used all Medicare Provider Analysis and Review files from January 1, 2011, to September 30, 2016, which contain nationwide encounter-level information about acute hospitalizations and inpatient rehabilitation facility (IRF) and skilled nursing facility (SNF) stays, as well as home health (HH) agency files to identify patients who received care for any of the 48 clinical episodes (24 medical and 24 surgical, defined by Medicare Severity–Diagnosis Related Groups [MS-DRGs]) included in the BPCI. We used the BPCI Analytic File to define bundled payment and nonbundled payment hospitals based on participation in BPCI Model 2, the largest of 4 models within the BPCI and basis for subsequent bundled payment programs.^32^ To mitigate complexities from time-varying participation, we treated all participating hospitals as participants after the quarter of contract initiation, regardless of subsequent dropout. We used the 2013-2016 MSSP beneficiary attribution files from the CMS to identify ACO-attributed beneficiaries, ie, individuals who were deemed to be patients of the ACO based on receiving the majority of their primary or specialty care through ACO clinicians. We created 2 beneficiary cohorts: attributed patients (ACO group) and nonattributed patients (non-ACO group).

We defined episodes per BPCI rules as hospitalization plus 90 days postdischarge, excluding individuals not continuously enrolled in Medicare fee-for-service as their primary payer for the care episode and a 180-day lookback period, those with end-stage kidney disease, those enrolled in Medicare Advantage, and those who died during the index hospitalization. We used 2011-2016 data from the American Hospital Association, Provider of Service File, Hospital Compare, and ACO Provider-level Research Identifiable Files to obtain hospital and ACO characteristics. We defined a baseline (pre–bundled payment) period before September 2013 and an intervention (bundled payment) period from October 1, 2013 (the start of the BPCI), to December 31, 2016. Within the bundled payment period, we defined participation based on the actual timing of BPCI contract initiation, thereby creating hospital-specific pre–bundled payment and bundled payment periods.

### Variables

The exposure variable in our analysis was a time-varying measure of hospitalization for a diagnosis, ie, MS-DRG, included in the BPCI from a hospital participating in the program for a clinical episode including that MS-DRG. The primary outcome was 90-day postdischarge institutional spending, which combined SNF, IRF, long-term acute care, and readmissions spending.

Secondary outcomes included 90-day unplanned readmissions; 90-day all-cause, all-location mortality; discharge to institutional postacute care (SNF or IRF); SNF length of stay (conditional on discharge to an SNF); and discharge with HH. Covariates included in models were patient-level (age, sex, race/ethnicity, disability status, dual eligibility for Medicare and Medicaid, and 29 Elixhauser clinical conditions) and time-varying market (ACO penetration, Medicare Advantage penetration, and number of Medicare beneficiaries) characteristics.^33,34^ Race and ethnicity data were obtained from Medicare claims, which contained White and Black categories that were used directly in analysis; the study team classified Asian, Hispanic, North American Native (including American Indian, Alaskan, Hawaiian, Samoan, and Guamanian natives), and other categories from claims data into the “Other” category. We also examined hospital-level (size, urban/rural location, ownership, and teaching status) and ACO-level (number of attributed beneficiaries, physicians, and postacute care facilities; inclusion of a hospital within the ACO) characteristics.

### Statistical Analysis

Data were analyzed between October 1, 2018, and June 10, 2021. We used a difference-in-differences method^35^ and ordinary least-squares regression to estimate differential changes in outcomes for bundled payment vs nonbundled payment patients in the pre–bundled payment vs bundled payment periods (which were hospital specific and time varying). To examine whether outcomes under bundled payments varied for patients attributed to an ACO, we also included an interaction between ACO attribution status and the bundled payment exposure variable (eMethods in the Supplement).

We used results from this regression model to estimate the changes in episode outcomes associated with hospitalization under bundled payments separately for ACO and non-ACO patients. We used Wald tests to evaluate whether differences in outcomes between the ACO and non-ACO patient groups were statistically different from 0 (ie, a statistical test on the significance of the interaction between the bundled payment exposure and patient ACO attribution status).^36^ In all analyses, we evaluated episodes for medical conditions (medical episodes) and surgeries or procedures (surgical episodes) separately because they involve different care processes that may have different associations with outcomes.^11,37,38^

We estimated models using 2013-2016 data to avoid measurement error in the exposure of interest, because ACO attribution was only available in CMS data starting in 2013, a period that predated the start of the BPCI. This process allowed comparisons of changes among ACO patients before and after bundled payments given the staggered entry into the BPCI that peaked in late 2014 and 2015. All models used time and hospital fixed effects, and models for the ACO cohort followed prior work and included ACO fixed effects.^21^ By allowing each ACO and hospital to serve as a control for itself, ACO and hospital fixed effects enabled examination associations within ACOs (ie, for bundled payment vs non–bundled payment patients attributed to the same ACO) and within hospitals (ie, for non-ACO vs ACO patients admitted to the same bundled payment hospital). We adopted this approach to mitigate bias from unobserved differences between ACOs and hospitals.

Models also used MS-DRG fixed effects to account for potential shifts between MS-DRGs within a given episode over time. For models estimating the 90-day unplanned readmissions outcome, we also controlled for the number of days alive during the period after hospital discharge to account for the shorter exposure time for patients who died.

The assumption of parallel trends between bundled payment and non–bundled payment hospitals was examined before bundled payments (June 1, 2011, to September 30, 2013) for the combined non-ACO and ACO groups using similar regression models with an indicator for hospital–MS-DRG–specific participation in bundled payments, categorical time (quarter-year) indicator variables, and the interactions. We were unable to examine ACO vs non-ACO group trends during the pre–bundled payment period owing to a lack of data about ACO status. Wald tests did not indicate divergent pre–bundled payment trends for medical or surgical episodes for any outcome (eFigures 1 and 2 in the Supplement). Visual examination of raw trends also did not reveal obviously divergent baseline trends (eFigures 3 and 4 in the Supplement).

We tested the robustness of our results via sensitivity analyses. We repeated analyses (1) using generalized linear models with log link and gamma distribution to test for sensitivity to model specification for the primary outcome, (2) excluding ACO fixed effects to allow for greater power in case within-ACO effects were underpowered and to remove imbalance from not having ACO fixed effects before the start of ACO participation, (3) excluding ACO fixed effects and controlling for the number of years of experience an ACO had in the MSSP model at the time of an episode, and (4) examining ACOs that included hospitals separately (distinguishing between ACOs that include vs do not include any hospital in their networks regardless of hospital bundled payment participation) to examine whether any observed associations were heavily influenced by ACOs with hospitals.

Statistical tests were 2-tailed and considered significant at α = .05. Robust SEs were corrected for heteroscedasticity and clustered at the hospital level. We analyzed complete case data. Analyses were performed using SAS, version 9.4 (SAS Institute Inc) and STATA, version 16.0 (StataCorp LLC).

## Results

A total of 7 108 146 beneficiaries (mean [SD] age, 76.9 [12.2] years; 4 101 081 women [58%]) received care for medical episodes (eTables 1-4 in the Supplement). A total of 3 675 962 beneficiaries (mean [SD] age, 74.8 [10.1] years; 2 074 921 women [56%]) received care for surgical episodes. Among patients who received care for medical episodes, 1 421 705 were ACO-attributed and 1 183 339 received care under bundled payments (eTable 2 in the Supplement). Among patients who received care for surgical episodes, 746 168 were ACO-attributed and 819 984 received care under bundled payments (eTable 4 in the Supplement).

In both non-ACO and ACO groups, there were small differences in patient age, sex, and clinical case-mix between patients receiving medical (Table 1; eTables 1 and 2 in the Supplement) and surgical (Table 2; eTables 3 and 4 in the Supplement) care via bundled payment programs and nonbundled payments. The number of ACOs in our sample increased from 220 in 2013 to 432 in 2016, with increases over time in the mean number of attributed patients and physicians and the proportion of ACOs that included a hospital (eTable 5 in the Supplement). Compared with non–bundled payment hospitals, bundled payment hospitals tended to be larger and more likely to be urban, nonprofit teaching hospitals (eTable 6 in the Supplement).

**Table 1.** Characteristics of Patients Admitted for Medical Episodes in the Pre–Bundled Payment Period by ACO Attribution and Bundled Payment Status, 2013 Quarter 1 to 2013 Quarter 3

Variable	Non-ACO group (n = 1 757 870)		ACO group (n = 220 514)	
	Nonbundled payment patients (n = 1 497 728)	Bundled payment patients (n = 260 142)	Nonbundled payment patients (n = 188 423)	Bundled payment patients (n = 32 091)
Age, mean (SD), y	77.2 (12.3)	77.6 (12.3)	77.9 (11.9)	78.1 (11.7)
Race and ethnicity, No. (%)				
Black	162 933 (10.9)	30 359 (11.7)	18 080 (9.6)	2844 (8.9)
Hispanic	30 113 (2.0)	5420 (2.1)	573 (1.8)	3423 (1.8)
White	1 255 169 (83.8)	216 258 (83.1)	160 412 (85.1)	27 723 (86.4)
Other^a^	49 513 (3.3)	8105 (3.1)	6508 (3.5)	951 (3.0)
Men, No. (%)	614 612 (41.0)	106 545 (41.0)	76 048 (40.4)	12 901 (40.2)
Women, No. (%)	883 116 (59.0)	153 597 (59.0)	112 375 (59.6)	19 190 (59.8)
Dual-eligible, No. (%)	402 461 (26.9)	62 559 (24.1)	44 710 (23.7)	6980 (21.8)
Residence in zip code, No. (%)				
Low income	388 991 (26.0)	51 844 (19.9)	37 282 (19.8)	5853 (18.2)
Low education	288 401 (19.3)	43 185 (16.6)	27 802 (14.7)	43 591 (14.3)
Elixhauser Comorbidity Index, mean (SD), points^b^	11.4 (11.4)^c^	11.6 (11.5)^d^	11.3 (11.4)^c^	11.4 (11.5)^c^
Most common clinical episodes, No. (%)				
Simple pneumonia and respiratory infections	163 852 (10.9)	25 647 (9.9)	19 839 (10.5)	3270 (10.2)
Sepsis	142 415 (9.5)	25 985 (10.0)	16 941 (9.0)	3172 (9.9)
Chronic obstructive pulmonary disease, bronchitis, or asthma	131 485 (8.8)	20 817 (8.0)	16 035 (8.5)	2471 (7.7)
Congestive heart failure	112 065 (7.5)	19 855 (7.6)	14 319 (7.6)	2491 (7.8)
Cardiac arrhythmia	89 184 (6.0)	16 330 (6.3)	12 034 (6.4)	2088 (6.5)
Stroke	83 909 (5.6)	15 566 (6.0)	10 033 (5.3)	1875 (5.8)
Urinary tract infection	83 346 (5.6)	14 311 (5.5)	10 513 (5.6)	1759 (5.5)
Kidney failure	79 244 (5.3)	14 135 (5.4)	10 035 (5.3)	1702 (5.3)
Esophagitis, gastroenteritis, and other digestive disorders	76 929 (5.1)	13 369 (5.1)	10 263 (5.4)	1708 (5.3)
Gastrointestinal hemorrhage	67 208 (4.5)	12 309 (4.7)	8777 (4.7)	1550 (4.8)
Most common comorbidities, No. (%)				
Hypertension	1 202 791 (80.3)	209 294 (80.5)	153 768 (81.6)	26 215 (81.7)
Fluid and electrolyte disorders	679 761 (45.4)	119 681 (46.0)	84 346 (44.8)	14 478 (45.1)
Chronic lung disease	558 123 (37.3)	91 542 (35.2)	68 520 (36.4)	11 406 (35.5)
Diabetes	478 342 (31.9)	81 308 (31.3)	59 292 (31.5)	9932 (31.0)
Congestive heart failure	462 012 (30.9)	79 265 (30.5)	58 271 (30.9)	9936 (31.0)

Abbreviation: ACO, accountable care organization.

^a^ Includes Asian, North American Native, and Other categories as reported in the Medicare claims data.

^b^ Mean and SD were reported because the Elixhauser Comorbidity Index is a variable included in regression models, producing a score that varies from −19 to 89 points, with larger values corresponding to higher mortality risk.

^c^ Mean (interquartile range) reported due to skewness, 10.0 (2.0-18.0).

^d^ Mean (interquartile range) reported due to skewness, 10.0 (2.0-19.0).

**Table 2.** Characteristics of Patients Admitted for Surgical Episodes in the Pre–Bundled Payment Period by ACO Attribution and Bundled Payment Status, 2013 Quarter 1 to 2013 Quarter 3

Variable	Non-ACO group (n = 738 690)		ACO group (n = 95 092)	
	Nonbundled payment patients (n = 585 043)	Bundled payment patients (n = 153 647)	Nonbundled payment patients (n = 70 208)	Bundled payment patients (n = 24 884)
Age, mean (SD), y	74.8 (10.1)	74.8 (10.0)	75.2 (9.8)	75.0 (9.7)
Race and ethnicity, No. (%)				
Black	36 317 (6.2)	11 237 (7.3)	4038 (5.8)	1438 (5.8)
Hispanic	7203 (1.2)	1864 (1.2)	740 (1.1)	228 (0.9)
White	525 599 (89.8)	136 061 (88.6)	63 489 (90.4)	22 510 (90.5)
Other^a^	15 924 (2.7)	4485 (2.9)	1941 (2.8)	708 (2.9)
Men, No. (%)	251 344 (43.0)	67 019 (43.6)	29 777 (42.4)	10 743 (43.2)
Women, No. (%)	333 699 (57.0)	86 628 (56.4)	40 431 (57.6)	14 141 (56.8)
Dual-eligible, No. (%)	92 495 (15.8)	22 506 (14.7)	9473 (13.5)	3169 (12.7)
Residence in zip code, %				
Low income	130 092 (22.2)	28 864 (18.8)	11 726 (16.7)	3925 (15.8)
Low education	89 093 (15.2)	21 875 (14.2)	7835 (11.2)	2833 (11.4)
Elixhauser Comorbidity Index, mean (SD), points^b^^,^^c^	4.1 (9.1)	4.2 (9.3)	4.0 (9.2)	4.0 (9.1)
Most common clinical episodes, No. (%)				
Major joint replacement of the lower extremity	184 299 (31.5)	46 652 (30.4)	22 898 (32.6)	7911 (31.8)
Percutaneous coronary intervention	62 554 (10.7)	18 016 (11.7)	7496 (10.7)	2900 (11.7)
Hip and femur procedures except major joint	51 331 (8.8)	11 350 (7.4)	5777 (8.2)	1798 (7.2)
Major bowel procedure	39 038 (6.7)	9571 (6.2)	4824 (6.9)	1539 (6.2)
Spinal fusion (noncervical)	29 391 (5.0)	7797 (5.1)	3367 (4.8)	1231 (4.9)
Pacemaker	24 479 (4.2)	6572 (4.3)	3136 (4.5)	1043 (4.2)
Other vascular surgery	23 584 (4.0)	6226 (4.1)	2734 (3.9)	931 (3.7)
Cardiac valve	19 213 (3.3)	7155 (4.7)	2330 (3.3)	1185 (4.8)
Coronary artery bypass graft	18 924 (3.2)	5237 (3.4)	2070 (2.9)	870 (3.5)
Major joint replacement of the upper extremity	16 526 (2.8)	4204 (2.7)	2134 (3.0)	689 (2.8)
Most common comorbidities, No. (%)				
Hypertension	450 391 (77.0)	118 830 (77.3)	54 765 (78.0)	19 309 (77.6)
Diabetes	147 404 (25.2)	38 844 (25.3)	17 541 (25.0)	6004 (24.1)
Chronic lung disease	126 187 (21.6)	32 874 (21.4)	14 815 (21.1)	5319 (21.4)
Hypothyroidism	112 071 (19.2)	28 994 (18.9)	13 529 (19.3)	4712 (18.9)
Obesity	87 725 (15.0)	24 516 (16.0)	10 740 (15.3)	3817 (15.3)

Abbreviation: ACO, accountable care organization.

^a^ Includes Asian, North American Native, and Other categories as reported in the Medicare claims data.

^b^ Mean and SD were reported because the Elixhauser Comorbidity Index is a variable included in regression models, producing a score that varies from −19 to 89 points, with larger values corresponding to higher mortality risk.

^c^ Mean (interquartile range) reported due to skewness, 0.0 (–1.0 to 8.0).

### Medical Episodes

In the pre–bundled payment period (eTable 7 in the Supplement) among patients not attributed to an ACO, those in bundled payment programs had higher postdischarge institutional spending ($6571) than those not in bundled payment programs ($6135). Similarly, among patients attributed to an ACO, postdischarge institutional spending for those in bundled payment programs was $6479 in the pre–bundled payment period compared with $6328 among those not in bundled payment programs. In both non-ACO and ACO groups, there were small differences in secondary outcome rates between patients in bundled payment programs and those not in bundled payment programs.

**Figure 1.**
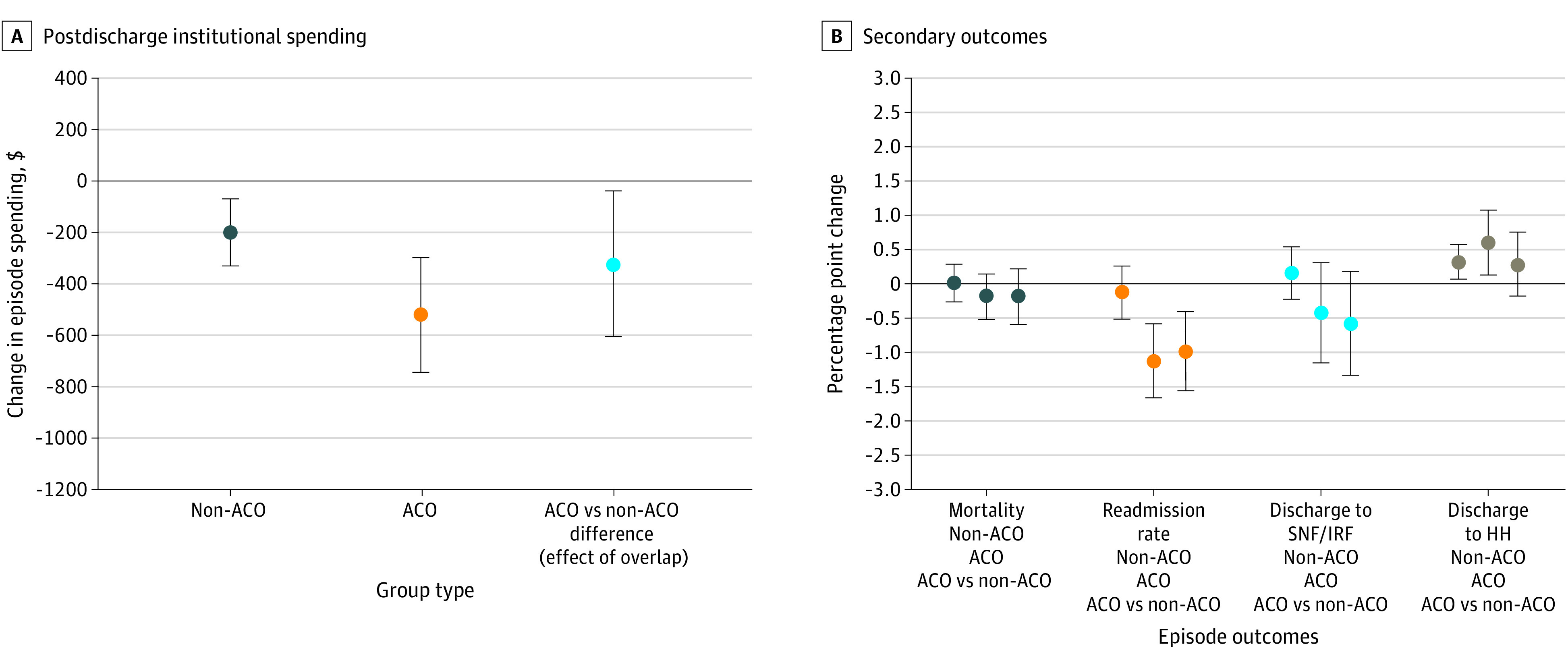
Changes in Medical Episode Outcomes Associated With Bundled Payments Among Non–Accountable Care Organization (ACO) and ACO Groups, 2013-2016 Risk-adjusted results from a difference-in-differences model using ACO, hospital, Medicare Severity-Diagnosis Related Groups (MS-DRGs), and time-fixed effects in postdischarge institutional spending (A) and secondary outcomes (B). A negative change in spending reflects savings associated with overlap. Error bars indicate 95% CI, and values are in US dollars. HH indicates home health; IRF, inpatient rehabilitation facility; and SNF, skilled nursing facility.

In the non-ACO group, patients in bundled payment programs had $200 differentially lower postdischarge institutional spending (95% CI, –$329 to –$70; *P* = .003 or a –5.1% change) than patients not in bundled payment programs (Figure 1A; eTable 8 in the Supplement). Similarly, in the ACO group, patients in bundled payment programs also had differentially lower spending (difference-in-differences, –$522; 95% CI, –$746 to –$299; *P* < .001 or a –8.23% change) than patients not in bundled payment programs. Compared with those in the non-ACO group, patients in bundled payment programs in the ACO group had greater decreases in postdischarge institutional spending (difference of –$323; 95% CI, –$607 to –$39; *P* = .03 or a –3.34 percentage point difference) (Figure 1A).

With respect to secondary outcomes, in the non-ACO group, there were differential differences in discharge to HH (0.31 percentage point higher for patients in bundled payment programs; 95% CI, 0.06-0.56; *P* = .02) (Figure 1B) and SNF length of stay (0.4 day lower for patients in bundled payment programs; 95% CI, –0.6 to –0.2; *P* < .001) (eFigure 5 in the Supplement). In the ACO group, patients in bundled payment programs had differentially lower 90-day readmissions (−1.1 percentage points; 95% CI, –1.70 to –0.59; *P* < .001), higher discharge to HH (0.58 percentage point; 95% CI, 0.11 to 1.06; *P* = .02), and lower SNF length of stay (−0.9 day; 95% CI, –1.2 to –0.6; *P* < .001) than patients not in bundled payment programs (Figure 1B; eFigure 5 in the Supplement).

Compared with patients in bundled payment programs in the non-ACO group, patients in bundled payment programs in the ACO group who received care for medical episodes had greater decreases in SNF length of stay (difference of −0.5 day; 95% CI, –0.9 to –0.1; *P* = .01) (eFigure 5 in the Supplement) and 90-day unplanned readmissions (difference of −0.98 percentage point; 95% CI, –1.55 to –0.41; *P* = .001) (Figure 1B).

### Surgical Episodes

In the pre–bundled payment period (eTable 9 in the Supplement), postdischarge institutional spending did not exhibit large differences for patients in bundled payment programs and not in bundled payment programs in the non-ACO group ($6376 and $6317, respectively) and the ACO group ($6034 and $6509, respectively). In both non-ACO and ACO groups, pre–bundled payment rates of secondary outcomes also varied with small differences across patients in bundled payment programs and not in bundled payment programs.

**Figure 2.**
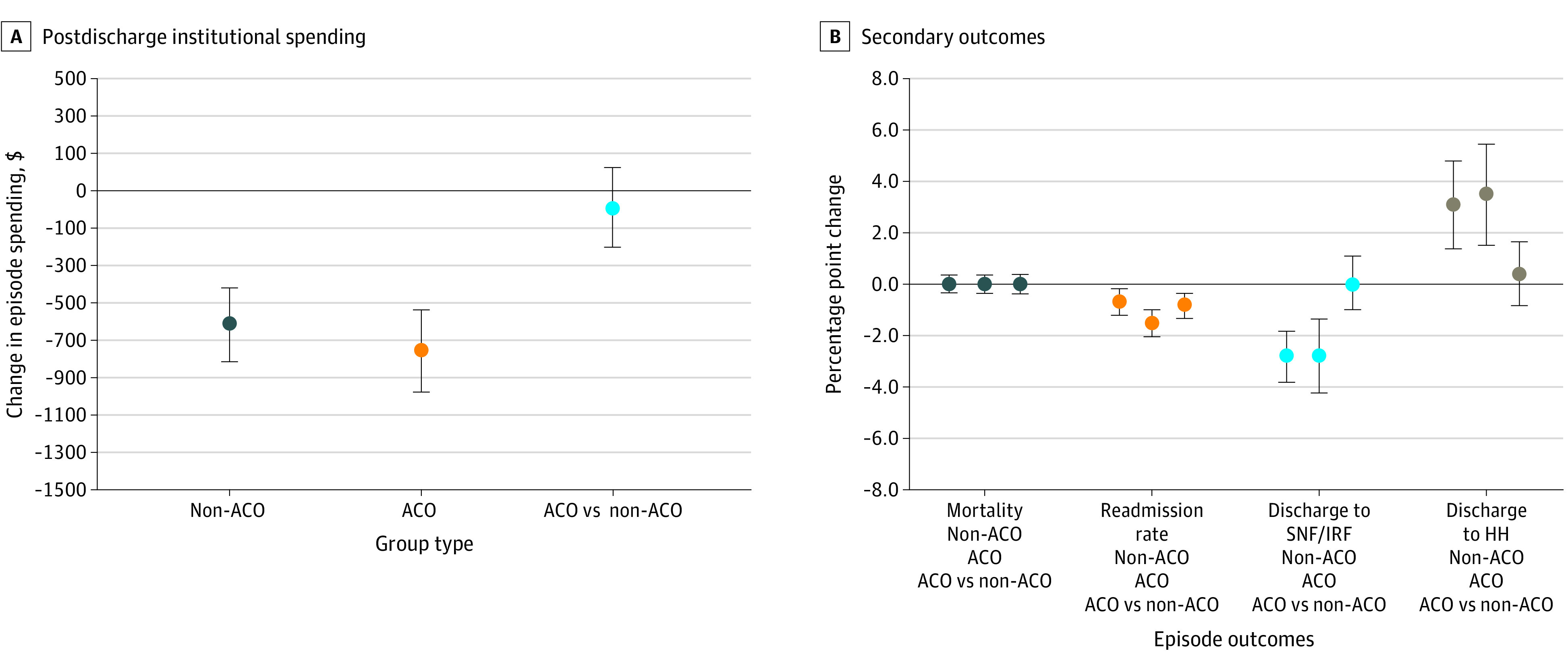
Changes in Surgical Episode Outcomes Associated With Bundled Payments Among Non–Accountable Care Organization (ACO) and ACO Groups, 2013-2016 Risk-adjusted results from a difference-in-differences model using ACO, hospital, Medicare Severity-Diagnosis Related Groups (MS-DRGs), and time-fixed effects in postdischarge institutional spending (A) and secondary outcomes (B). A negative change in spending reflects savings associated with overlap. Error bars indicate 95% CI, and values are in US dollars. HH indicates home health; IRF, inpatient rehabilitation facility; and SNF, skilled nursing facility.

In the non-ACO group, patients in bundled payment programs had differentially lower postdischarge institutional spending than patients not in bundled payment programs (difference-in-differences, –$693; 95% CI, –$870 to –$517; *P* < .001 or a −11.0% change) (Figure 2A). In the ACO group, patients in bundled payment programs had differentially lower postdischarge institutional spending compared with patients not in bundled payment programs (difference-in-differences, –$817; 95% CI, –$1014 to –$620; *P* < .001 or a −12.9% change) (Figure 2A). Patients in bundled payment programs in the ACO and non-ACO groups did not differ with respect to changes in spending (difference of –$124; 95% CI, –$316 to $68; *P* = .21 or a −1.9 percentage point difference) (Figure 2A).

Among patients not attributed to ACOs, differential differences for secondary outcomes demonstrated comparatively lower 90-day readmissions (difference of –0.69 percentage point; 95% CI, –$1.21 to –$0.17; *P* = .009), lower discharge to institutional postacute care (difference of –2.82 percentage points; 95% CI, –$3.81 to –$1.82; *P* < .001), higher discharge to HH (difference of 3.10 percentage points; 95% CI, 1.39 to 4.80; *P* < .001), and shorter SNF length of stay (–1.12 days; 95% CI, –1.37 to –0.88; *P* < .001) for patients in bundled payment programs (Figure 2B; eFigure 6 in the Supplement). Similarly, in the ACO group, patients in bundled payment programs had lower 90-day readmissions (difference of –1.53 percentage points; 95% CI, –$2.04 to –$1.01; *P* < .001), fewer instances of discharge to institutional postacute care (difference of –2.78 percentage points; 95% CI, –$4.23 to –$1.34; *P* < .001), reduced SNF length of stay (–1.21 days; 95% CI, –1.51 to 0.90; *P* < .001), and higher discharge to HH (difference of 3.5 percentage points; 95% CI, 1.52 to 5.47; *P* = .001) than patients not in bundled payment programs (Figure 2B; eFigure 6 in the Supplement).

Patients in bundled payment programs in the ACO group had greater decreases in 90-day unplanned readmissions (difference-in-differences, −0.84 percentage point; 95% CI, −1.32 to −0.35; *P* = .001) than patients in bundled payment programs in the non-ACO group (Figure 2B). In contrast, there were no differences in changes in SNF length of stay, mortality, discharge to institutional postacute care, and discharge with HH for surgical episodes (Figure 2B; eFigure 6 in the Supplement).

### Sensitivity Analyses

Results were qualitatively similar from analyses excluding ACO fixed effects (eFigures 7 and 8 in the Supplement), excluding ACO fixed effects and controlling for the number of years of experience an ACO had in the MSSP (eFigures 9 and 10 in the Supplement), and using generalized linear rather than ordinary least squares models (eFigures 11 and 12 in the Supplement). Restricting the sample to ACOs including a hospital did not produce results with stronger, or similar, differences in outcomes by non-ACO vs ACO group compared with the main analysis (eTables 10 and 11 in the Supplement).

## Discussion

In this cohort study, compared with inclusion in bundled payments alone, simultaneous inclusion in both ACOs and bundled payments was associated with lower institutional postacute care spending and readmissions for medical episodes and with lower readmissions but not lower spending for surgical episodes. Our study results suggest 3 possible policy implications.

First, our results suggest the potential additive benefits when bundled payments and other payment arrangements overlap. In particular, we observed improvements in terms of both higher quality (eg, a 1–percentage point differential decrease in readmissions) and lower spending (eg, a 5% decrease in postdischarge institutional spending per medical episode). It is particularly notable that quality improvements were observed for both surgical and medical episodes given that both episode types have been associated with gross savings to Medicare without changes in quality.^11,39^

The comparatively better outcomes may reflect partially additive benefits of patients receiving complementary practice redesign services under different payment models, such as ACOs driving broader investments in ambulatory care infrastructure and processes that reduce hospitalization, and bundled payment participants focusing more specifically on reducing costly institutional postacute care. Anecdotally, some organizations have adopted this approach, using ACOs and bundled payments in concert to achieve performance that may be more difficult using 1 payment model alone. These findings suggest that policy makers could continue promoting both ACOs and bundled payments, encouraging added benefit by enabling patients to receive care through multiple models simultaneously.

Second, our results suggest that policy makers may benefit from revisiting the existing approach for handling ACO-bundle overlap. Currently, when an ACO’s attributed beneficiaries receive care from another organization outside the ACO for care covered by bundled payments, that outside organization’s historical episode spending is counted against the ACO. This approach effectively penalizes ACOs whose patients receive care from rapidly improving bundled payment participants.^28,40^

These are policy-relevant points given that, despite these unfavorable dynamics, we found that ACO patients nonetheless benefited from receiving care at bundled payment hospitals. Given our finding that ACOs appear to enhance episodic care, the CMS should encourage overlap of payment arrangements. To accomplish this outcome, ACOs could be allowed to share in, rather than be penalized for, the benefits associated with overlapping care. The potential for additive payment model benefits may be even greater under policy approaches that more directly encourage overlap and better recognize the contributions of both ACO and bundled payment participants in driving episode outcomes.

Third, our results highlight the need for more work to evaluate outcomes when different payment models overlap in patients’ care. This issue is especially important as situations of overlap increase. For instance, in the US, these situations can occur with simultaneous implementation of large payment models in primary care (eg, Comprehensive Primary Care Plus), specialty care (eg, Oncology Care Model), and their combination (eg, newer ACO or Direct Contracting).

Our study used bundled payments to provide the first evidence about overlapping payment models. The results can counterbalance policy maker concerns and even encourage policies that permit these dynamics in some cases. As various payment model types proliferate, policy makers and practice leaders would benefit from insight about situations in which overlap is or is not beneficial.

### Limitations

This analysis has some limitations. First, findings may have been subject to residual confounding and selection bias. However, we mitigated these concerns by using a difference-in-differences design that incorporated fixed effects to account for unobserved heterogeneity and directly accounted for multiple patient and hospital characteristics. Second, given the lack of ACO attribution in the preperiod and time-varying nature of participation, our analysis to inform the parallel trends assumption could not definitively rule out pretrends, which were more apparent for surgical episodes. Third, we included only 1 ACO program in our analysis. However, the MSSP is the largest and longest running nationwide ACO program to date in the US.

Fourth, we evaluated outcomes under the BPCI as a single bundled payment program. However, BPCI Model 2 captured a wide range of procedures and conditions in the 48 episodes and served as the basis for subsequent models, such as BPCI Advanced. Fifth, we did not evaluate how inclusion in or exclusion from bundled payments affected the relationship between ACO participation and outcomes. However, given the focused scope of bundled payments on 90-day episodes, the relationships related to overlapping care are likely most visible in the episode-based occurrences evaluated in our analysis.

## Conclusions

The results of this cohort study suggest that, among hospitalized patients, ACO attribution was associated with changes that reflected comparatively greater benefits associated with bundled payments. These findings suggest that policy makers and payment model participants could approach overlap more favorably when making policy and participation decisions.

## Notes

Supplement. **eMethods.** Model Specification **eFigure 1.** Adjusted Parallel Trends for Medical Episodes, 2011 Quarter 3 to 2013 Quarter 3 **eFigure 2.** Adjusted Parallel Trends for Surgical Episodes, 2011 Quarter 3 to 2013 Quarter 3 **eFigure 3.** Unadjusted Changes in Outcomes Associated With Bundled Payments Among Non-ACO and ACO-Attributed Patients Admitted for Medical Episodes, 2013 Quarter 1 to 2016 Quarter 3 **eFigure 4.** Unadjusted Changes in Outcomes Associated With Bundled Payments Among Non-ACO and ACO-Attributed Patients Admitted for Surgical Episodes, 2013 Quarter 1 to 2016 Quarter 3 **eTable 1.** Breakdown of Clinical Episodes by ACO Attribution and Bundled Payment Status **eTable 2.** Characteristics of Patients Admitted for Medical Episodes by Study Period, ACO Attribution, and Bundled Payment Status, 2013-2016 **eTable 3.** Characteristics of Patients Admitted for Surgical Episodes by ACO Attribution and Bundled Payment Status **eTable 4.** Characteristics of Patients Admitted for Surgical Episodes by Study Period, ACO Attribution, and Bundled Payment Status, 2013-2016 **eTable 5.** ACO Characteristics, 2013-2016 **eTable 6.** Hospital Characteristics by Bundled Payment Status, 2011-2013 **eTable 7.** Unadjusted Changes in Medical Episode Outcomes Prebundled and Postbundled Payments, by ACO Attribution **eTable 8.** Percent Changes in Medical and Surgical Episode Outcomes Associated With Bundled Payments Among Non-ACO and ACO-Attributed Patients, 2013 Quarter 1 to 2016 Quarter 3 **eFigure 5.** Changes in Medical Episode SNF Length of Stay Associated With Bundled Payments by ACO Attribution, 2013-2016 **eTable 9.** Unadjusted Changes in Surgical Episode Outcomes Prebundled and Postbundled Payments, by ACO Attribution, 2013-2016 **eFigure 6.** Changes in Surgical Episode SNF Length of Stay Associated With Bundled Payments by ACO Attribution, 2013-2016 **eFigure 7.** Sensitivity Analysis for Medical Episodes, Using Models Without ACO Fixed Effects, 2013-2016 **eFigure 8.** Sensitivity Analysis for Surgical Episodes, Using Models Without ACO Fixed Effects, 2013-2016 **eFigure 9.** Sensitivity Analysis for Medical Episodes Controlling for ACO Years of Experience, 2013-2016 **eFigure 10.** Sensitivity Analysis for Surgical Episodes Controlling for ACO Years of Experience, 2013-2016 **eFigure 11.** Sensitivity Analysis for Changes in Postdischarge Institutional Spending in Medical Episodes, Using Generalized Linear Models With Log Link and Gamma Distribution, 2013-2016 **eFigure 12.** Sensitivity Analysis for Changes in Postdischarge Institutional Spending in Surgical Episodes, Using Generalized Linear Models With Log Link and Gamma Distribution, 2013-2016 **eTable 10.** Sensitivity Analysis for Medical Episodes, ACOs Including Hospitals, 2013-2016 **eTable 11.** Sensitivity Analysis for Surgical Episodes, ACOs Including Hospitals, 2013-2016
