# Supplementary material for: Association of Patient Outcomes With Bundled Payments Among Hospitalized Patients Attributed to Accountable Care Organizations
Source: JAMA Health Forum. 2021 Aug 20;2(8):e212131. doi: 10.1001/jamahealthforum.2021.2131 (PMC8796940; doi:10.1001/jamahealthforum.2021.2131)
Supplement: Supplement. — eMethods. Model Specification eFigure 1. Adjusted Parallel Trends for Medical Episodes, 2011 Quarter 3 to 2013 Quarter 3 eFigure 2. Adjusted Parallel Trends for Surgical Episodes, 2011 Quarter 3 to 2013 Quarter 3 eFigure 3. Unadjusted Changes in Outcomes Associated With Bundled Payments Among Non-ACO and ACO-Attributed Patients Admitted for Medical Episodes, 2013 Quarter 1 to 2016 Quarter 3 eFigure 4. Unadjusted Changes in Outcomes Associated With Bundled Payments Among Non-ACO and ACO-Attributed Patients Admitted for Surgical Episodes, 2013 Quarter 1 to 2016 Quarter 3 eTable 1. Breakdown of Clinical Episodes by ACO Attribution and Bundled Payment Status eTable 2. Characteristics of Patients Admitted for Medical Episodes by Study Period, ACO Attribution, and Bundled Payment Status, 2013-2016 eTable 3. Characteristics of Patients Admitted for Surgical Episodes by ACO Attribution and Bundled Payment Status eTable 4. Characteristics of Patients Admitted for Surgical Episodes by Study Period, ACO Attribution, and Bundled Payment Status, 2013-2016 eTable 5. ACO Characteristics, 2013-2016 eTable 6. Hospital Characteristics by Bundled Payment Status, 2011-2013 eTable 7. Unadjusted Changes in Medical Episode Outcomes Prebundled and Postbundled Payments, by ACO Attribution eTable 8. Percent Changes in Medical and Surgical Episode Outcomes Associated With Bundled Payments Among Non-ACO and ACO-Attributed Patients, 2013 Quarter 1 to 2016 Quarter 3 eFigure 5. Changes in Medical Episode SNF Length of Stay Associated With Bundled Payments by ACO Attribution, 2013-2016 eTable 9. Unadjusted Changes in Surgical Episode Outcomes Prebundled and Postbundled Payments, by ACO Attribution, 2013-2016 eFigure 6. Changes in Surgical Episode SNF Length of Stay Associated With Bundled Payments by ACO Attribution, 2013-2016 eFigure 7. Sensitivity Analysis for Medical Episodes, Using Models Without ACO Fixed Effects, 2013-2016 eFigure 8. Sensitivity Analysis for Surgical Episodes, Using Models W [file jamahealthforum-e212131-s001.pdf]

Supplementary Online Content

Navathe AS, Liao JM, Wang E, et al. Association of patient outcomes with bundled payments among hospitalized patients attributed to accountable care organizations. *JAMA Health Forum*. 2021;2(8):e212131. doi:10.1001/jamahealthforum.2021.2131

eMethods. Model Specification

eFigure 1. Adjusted Parallel Trends for Medical Episodes, 2011 Quarter 3 to 2013 Quarter 3

eFigure 2. Adjusted Parallel Trends for Surgical Episodes, 2011 Quarter 3 to 2013 Quarter 3

eFigure 3. Unadjusted Changes in Outcomes Associated With Bundled Payments Among Non-ACO and ACO-Attributed Patients Admitted for Medical Episodes, 2013 Quarter 1 to 2016 Quarter 3

eFigure 4. Unadjusted Changes in Outcomes Associated With Bundled Payments Among Non-ACO and ACO-Attributed Patients Admitted for Surgical Episodes, 2013 Quarter 1 to 2016 Quarter 3

eTable 1. Breakdown of Clinical Episodes by ACO Attribution and Bundled Payment Status

eTable 2. Characteristics of Patients Admitted for Medical Episodes by Study Period, ACO Attribution, and Bundled Payment Status, 2013-2016

eTable 3. Characteristics of Patients Admitted for Surgical Episodes by ACO Attribution and Bundled Payment Status

eTable 4. Characteristics of Patients Admitted for Surgical Episodes by Study Period, ACO Attribution, and Bundled Payment Status, 2013-2016

eTable 5. ACO Characteristics, 2013-2016

eTable 6. Hospital Characteristics by Bundled Payment Status, 2011-2013

eTable 7. Unadjusted Changes in Medical Episode Outcomes Prebundled and Postbundled Payments, by ACO Attribution

eTable 8. Percent Changes in Medical and Surgical Episode Outcomes Associated With Bundled Payments Among Non-ACO and ACO-Attributed Patients, 2013 Quarter 1 to 2016 Quarter 3

eFigure 5. Changes in Medical Episode SNF Length of Stay Associated With Bundled Payments by ACO Attribution, 2013-2016

eTable 9. Unadjusted Changes in Surgical Episode Outcomes Prebundled and Postbundled Payments, by ACO Attribution, 2013-2016

© 2021 Navathe AS et al. *JAMA Health Forum.*

eFigure 6. Changes in Surgical Episode SNF Length of Stay Associated With Bundled Payments by ACO Attribution, 2013-2016

eFigure 7. Sensitivity Analysis for Medical Episodes, Using Models Without ACO Fixed Effects, 2013-2016

eFigure 8. Sensitivity Analysis for Surgical Episodes, Using Models Without ACO Fixed Effects, 2013-2016

eFigure 9. Sensitivity Analysis for Medical Episodes Controlling for ACO Years of Experience, 2013-2016

eFigure 10. Sensitivity Analysis for Surgical Episodes Controlling for ACO Years of Experience, 2013-2016

eFigure 11. Sensitivity Analysis for Changes in Postdischarge Institutional Spending in Medical Episodes, Using Generalized Linear Models With Log Link and Gamma Distribution, 2013-2016

eFigure 12. Sensitivity Analysis for Changes in Postdischarge Institutional Spending in Surgical Episodes, Using Generalized Linear Models With Log Link and Gamma Distribution, 2013-2016

eTable 10. Sensitivity Analysis for Medical Episodes, ACOs Including Hospitals, 2013-2016

eTable 11. Sensitivity Analysis for Surgical Episodes, ACOs Including Hospitals, 2013-2016

This supplementary material has been provided by the authors to give readers additional information about their work.

© 2021 Navathe AS et al. *JAMA Health Forum.*

eMethods. Model Specification

We used a difference-in-differences method and ordinary least squares regression to estimate differential changes in outcomes for bundled payment versus non-bundled payment patients in the pre-bundled payment versus bundled payment periods (which were hospital-specific and time-varying), including an interaction between ACO attribution status and the bundled payment exposure variable.

Specification: $Y_{i,h,t}\ (outcome) = \alpha_h + \beta_1 * BPCI\_now_{h,t} + \beta_2 * ACO_{i,t} + \gamma * ACO_{i,t} * BPCI\_now_{h,t} + \beta_3 * Quarter\_FE_t + \beta_4 * Hospital\_FE_h + \beta_5 * ACO\_FE_n + \theta * Xpt_{i,h,t} + M_{m,t} + e_{i,h,t}$.

BPCI_now was the time-varying indicator of hospital participation in BPCI, which together with hospital and time fixed effects, provided the difference-in-differences estimate for BPCI participation. *ACO* was an dichotomous indicator for a patient's attribution to an ACO (attributed to ACO = 1). The interaction term *BPCI_now * ACO* gave the differential effect of BPCI for ACO-attributed versus non-ACO patients' outcomes – the effect of interest. *ACO_FE* was a fixed effect for each ACO. *Xpt* was a vector of patient characteristics including age, sex, race/ethnicity, disability status, dual eligibility for Medicare and Medicaid, and 29 Elixhauser clinical conditions. *M* was a vector of time-varying market characteristics including ACO penetration, Medicare Advantage penetration, and number of Medicare beneficiaries. Note that because we use hospital fixed effects, this was a smaller unit than market fixed effects (which would be subsumed in the hospital fixed effect), so our model gave within-market (i.e., within-hospital) estimates.

We used results from this regression model to estimate the changes in episode outcomes associated with hospitalization under bundled payments separately for ACO and non-ACO patients. We used Wald tests to evaluate whether differences in outcomes between the ACO-attributed and non-ACO patient groups were statistically different from zero (i.e., a statistical test on the significance of the interaction between the bundled payments exposure and patient ACO attribution status [γ in equation above]).[40] In all analyses, we evaluated episodes for medical conditions (medical episodes) and surgeries or procedures (surgical episodes) separately because they involve different care processes that may have different effects on outcomes.

© 2021 Navathe AS et al. *JAMA Health Forum.*

eFigure 1. Adjusted Parallel Trends for Medical Episodes, 2011 Quarter 3 to 2013 Quarter 3

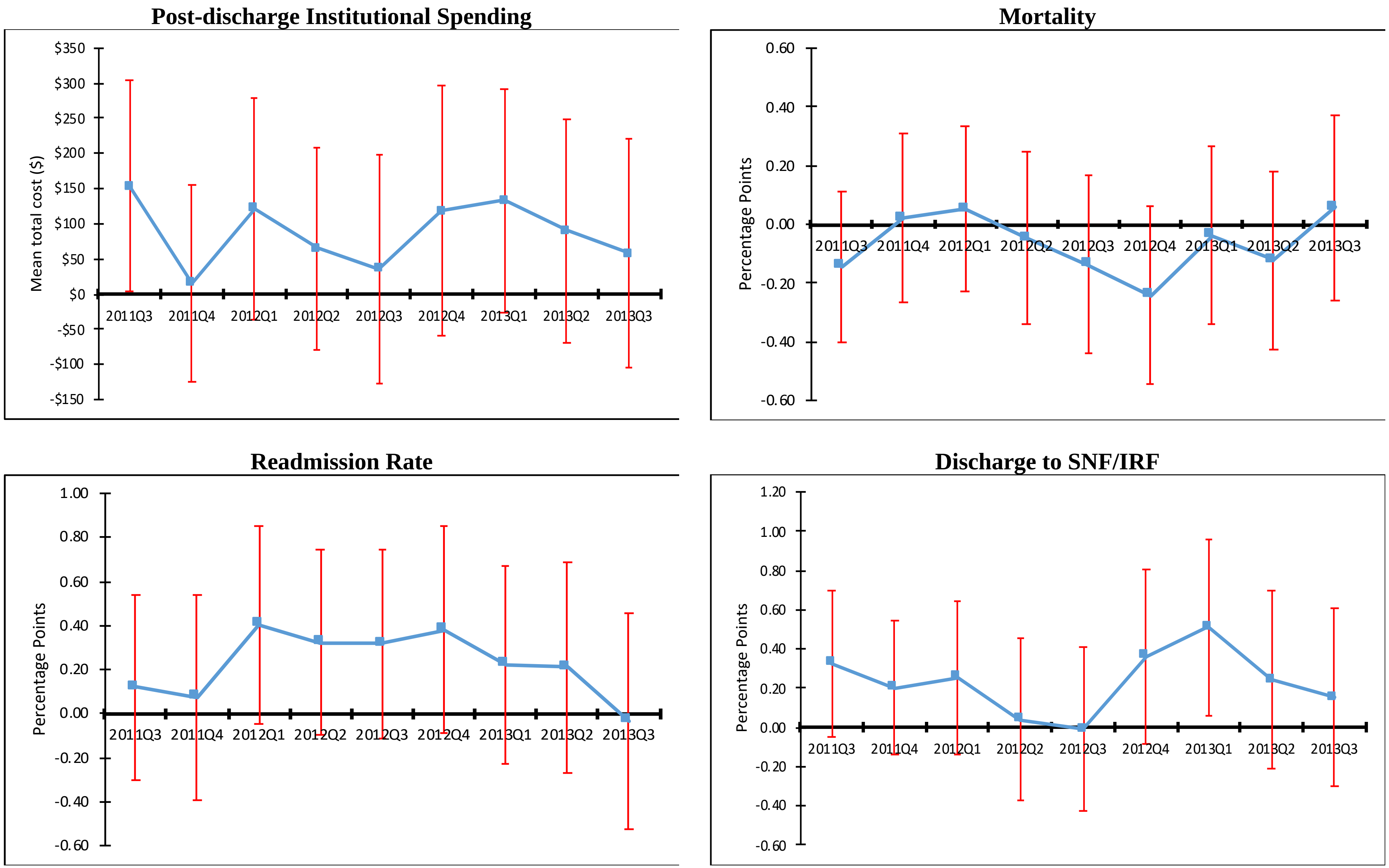

© 2021 Navathe AS et al. *JAMA Health Forum.*

Discharge to HH

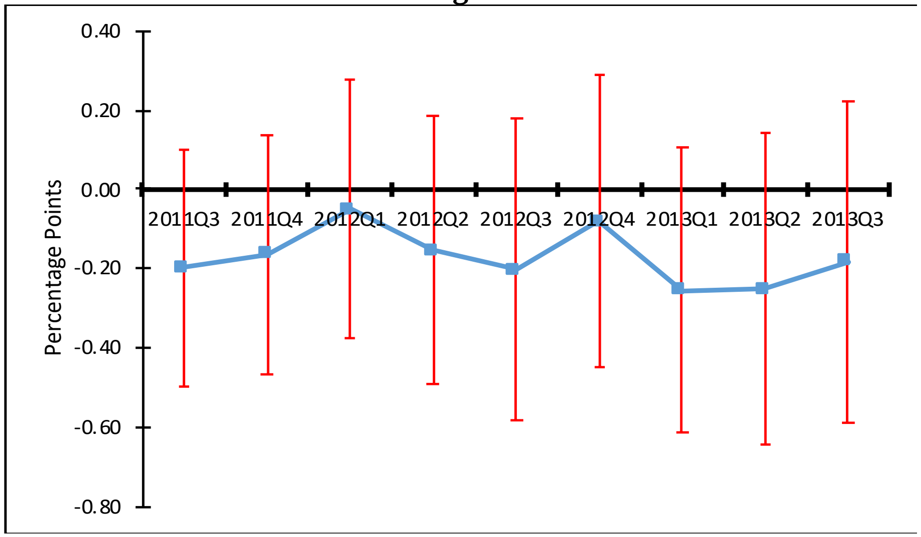

SNF Length of Stay

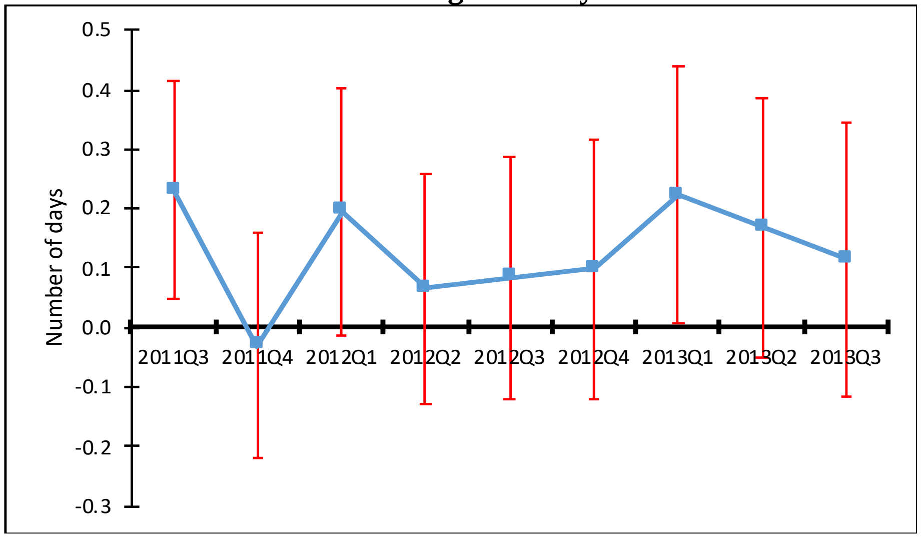

This set of figures shows trends in study outcomes for medical episodes in the pre-bundled payment period (2011 Q3 to 2013 Q3) comparing differences in outcomes for BPCI hospitals versus non-BPCI hospitals. We did not examine pre-ACO trends because this data was not available. The persistent, non-significant gap in the medical episodes is suggestive that on average BPCI hospitals had higher post-discharge institutional spending in the pre-period, but that there was no systematic trend toward closing that gap. The other outcomes look generally similar, though the readmission rate does show a decreasing trend in the last 4 quarters, although with overlapping confidence intervals with quarters earlier in the pre-period. Together, these figures provide supporting evidence for the parallel trends assumption under a difference-in-differences study design. **Abbreviations**: SNF, skilled nursing facility; IRF, inpatient rehabilitation facility; HH, home health agency.

© 2021 Navathe AS et al. *JAMA Health Forum.*

eFigure 2. Adjusted Parallel Trends for Surgical Episodes, 2011 Quarter 3 to 2013 Quarter 3

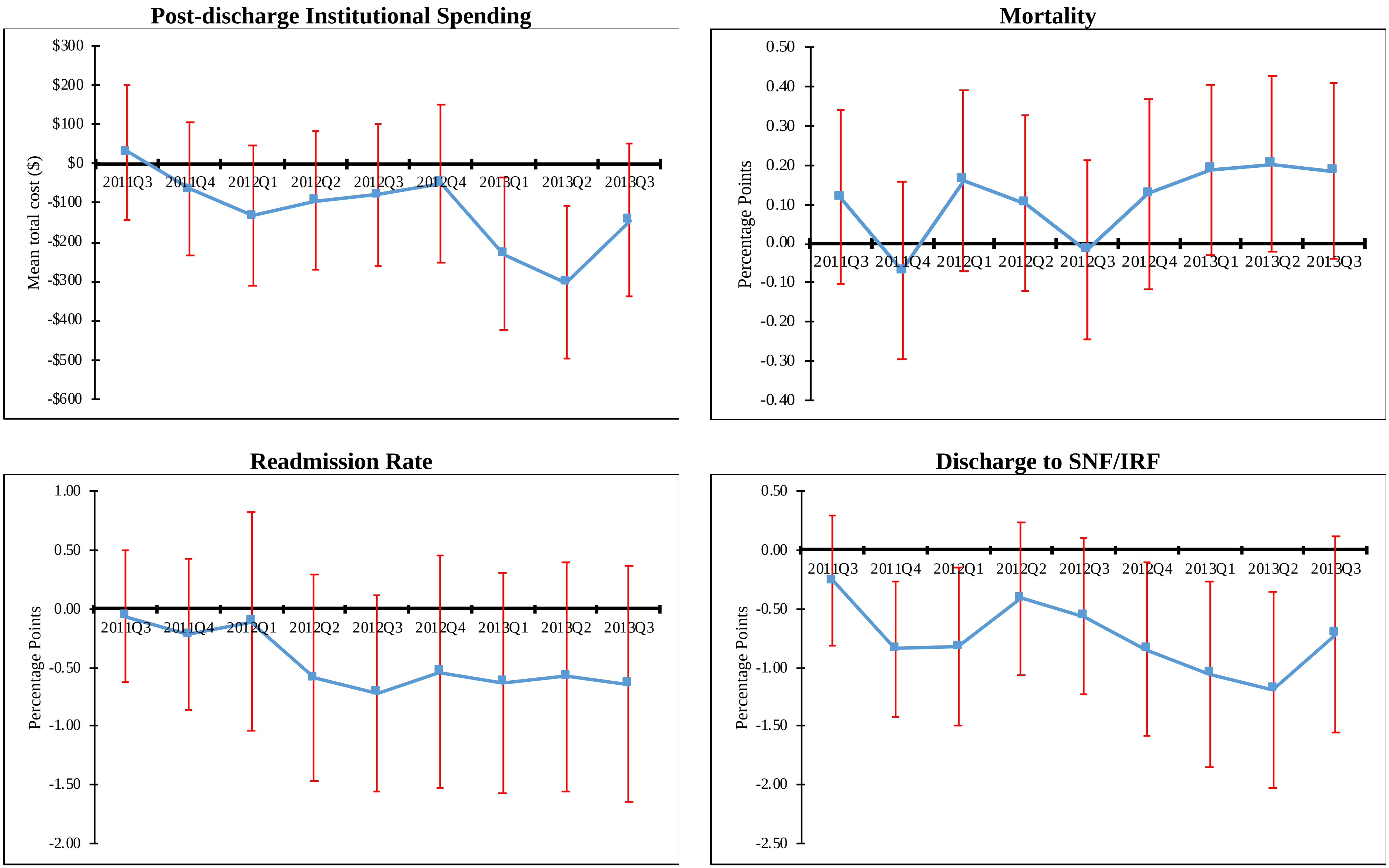

© 2021 Navathe AS et al. *JAMA Health Forum.*

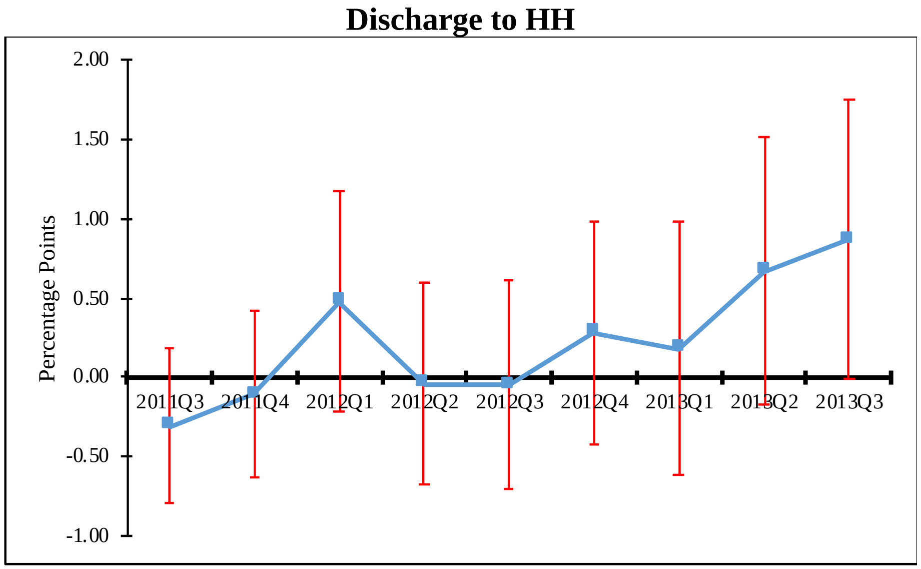

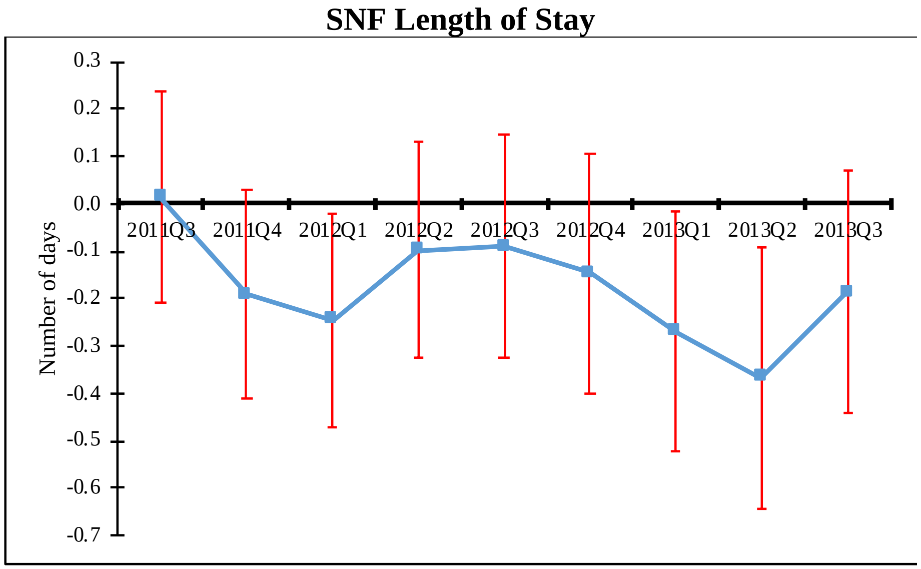

This set of figures shows trends in study outcomes for surgical episodes in the pre-bundled payment period (2011 Q3 to 2013 Q3), comparing differences in outcomes for BPCI hospitals versus non-BPCI hospitals. We did not examine pre-ACO trends because this data was not available. The parallel trends analysis for surgical episodes may be more suggestive of pre-trends, including for the post-discharge institutional spending discharge to SNF/IRF and HH, and SNF Length of Stay outcomes, although not definitively so. These estimates do not generally reach significance and have overlapping confidence intervals with estimates earlier in the pre-periods. Together, these figures providing supporting evidence for the parallel trends assumption under a difference-in-differences study design, although with some caution in interpretation given some suggestion of pre-trends.

Abbreviations: SNF, skilled nursing facility; IRF, inpatient rehabilitation facility; HH, home health agency.

© 2021 Navathe AS et al. *JAMA Health Forum.*

eFigure 3. Unadjusted Changes in Outcomes Associated With Bundled Payments Among Non-ACO and ACO-Attributed Patients Admitted for Medical Episodes, 2013 Quarter 1 to 2016 Quarter 3

Panel A: Number of Episodes

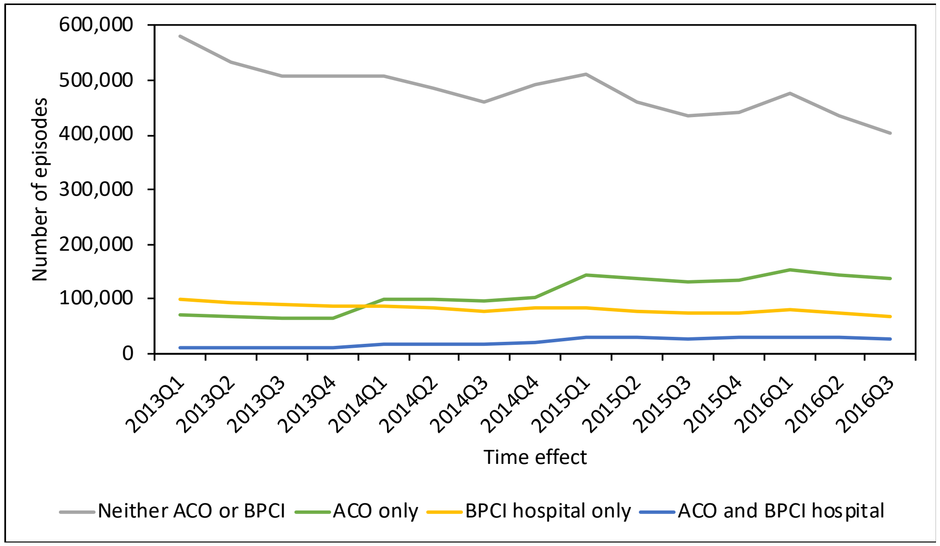

Panel B: Post-discharge Institutional Spending

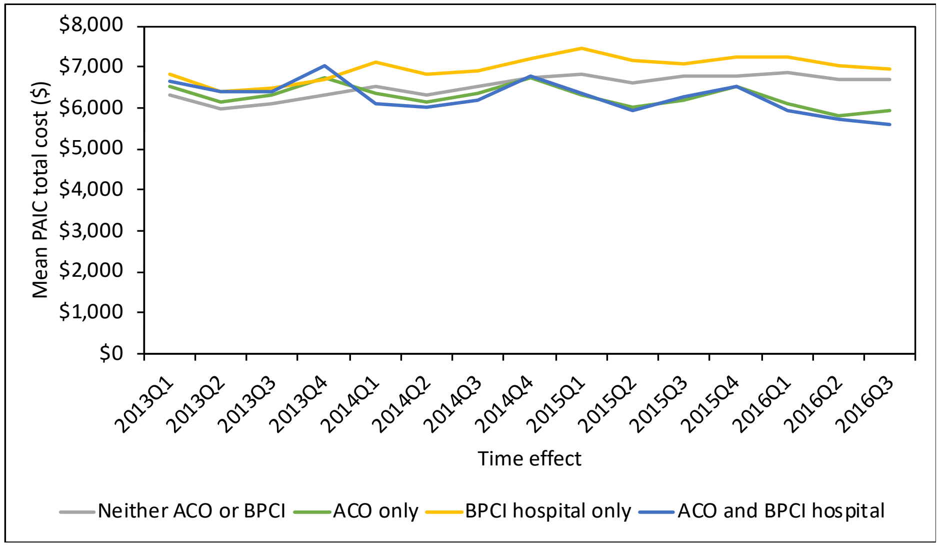

© 2021 Navathe AS et al. *JAMA Health Forum.*

Panel C: Quality and Utilization

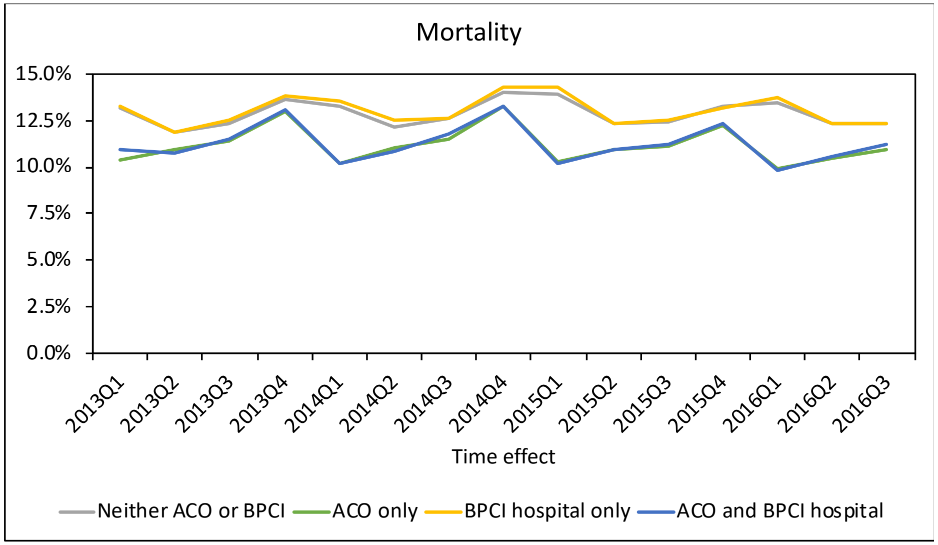

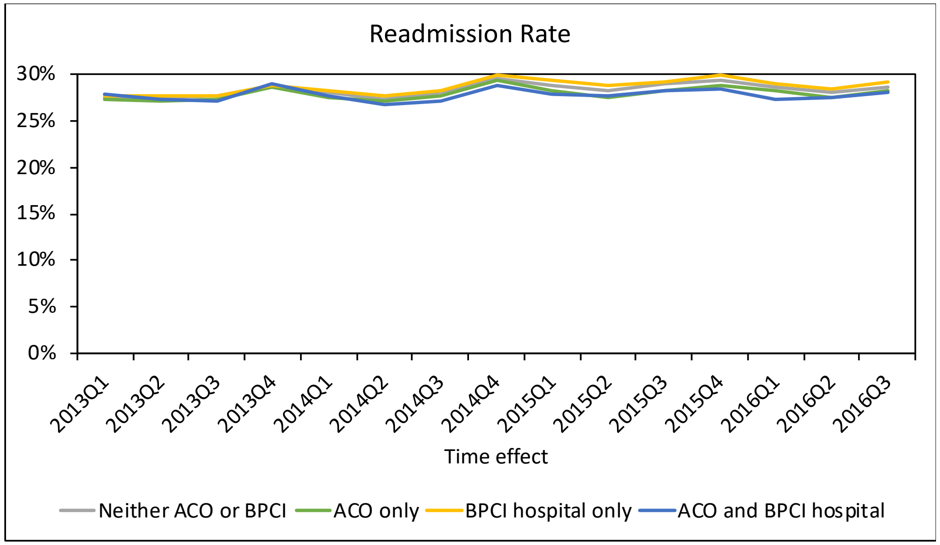

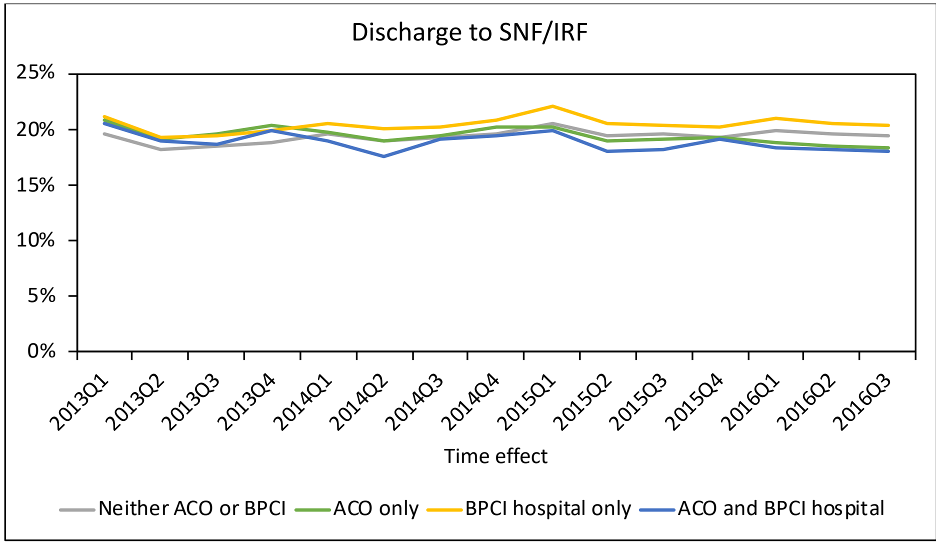

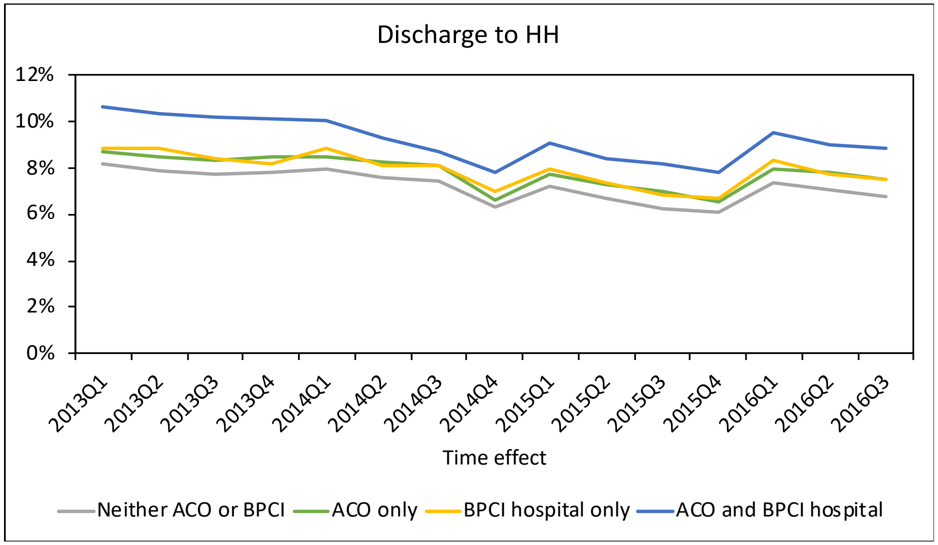

© 2021 Navathe AS et al. *JAMA Health Forum.*

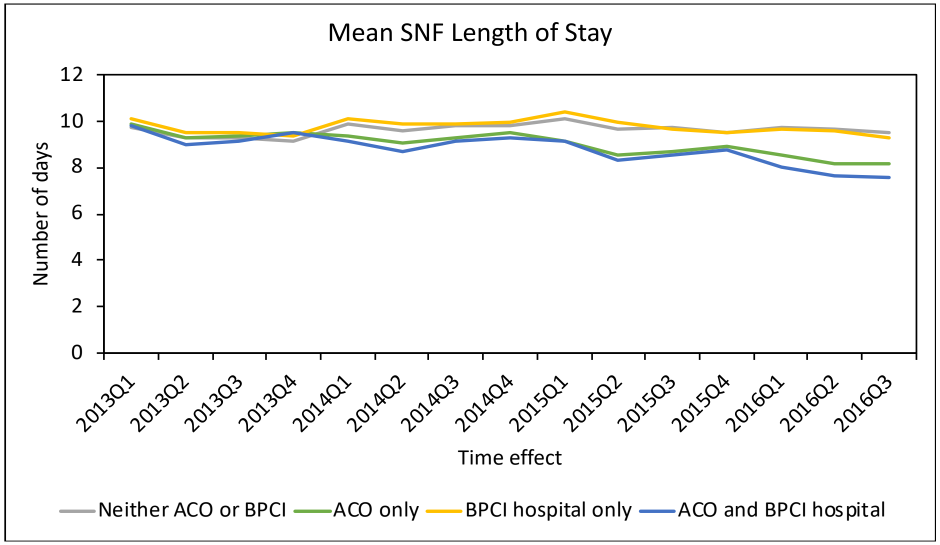

This set of figures shows unadjusted changes in study outcomes for medical episodes between 2013 Q1 to 2016 Q3 by hospital group: within the ACO group, ACO and BPCI hospitals and ACO only hospitals; and within the Non-ACO group, BPCI only hospitals and Neither ACO or BPCI hospitals. Importantly, these figures do not reflect pre-ACO time periods.

Abbreviations: ACO, accountable care organization; BPCI, Bundled Payments for Care Improvement; SNF, skilled nursing facility; IRF, inpatient rehabilitation facility; HH, home health agency.

© 2021 Navathe AS et al. *JAMA Health Forum.*

eFigure 4. Unadjusted Changes in Outcomes Associated With Bundled Payments Among Non-ACO and ACO-Attributed Patients Admitted for Surgical Episodes, 2013 Quarter 1 to 2016 Quarter 3

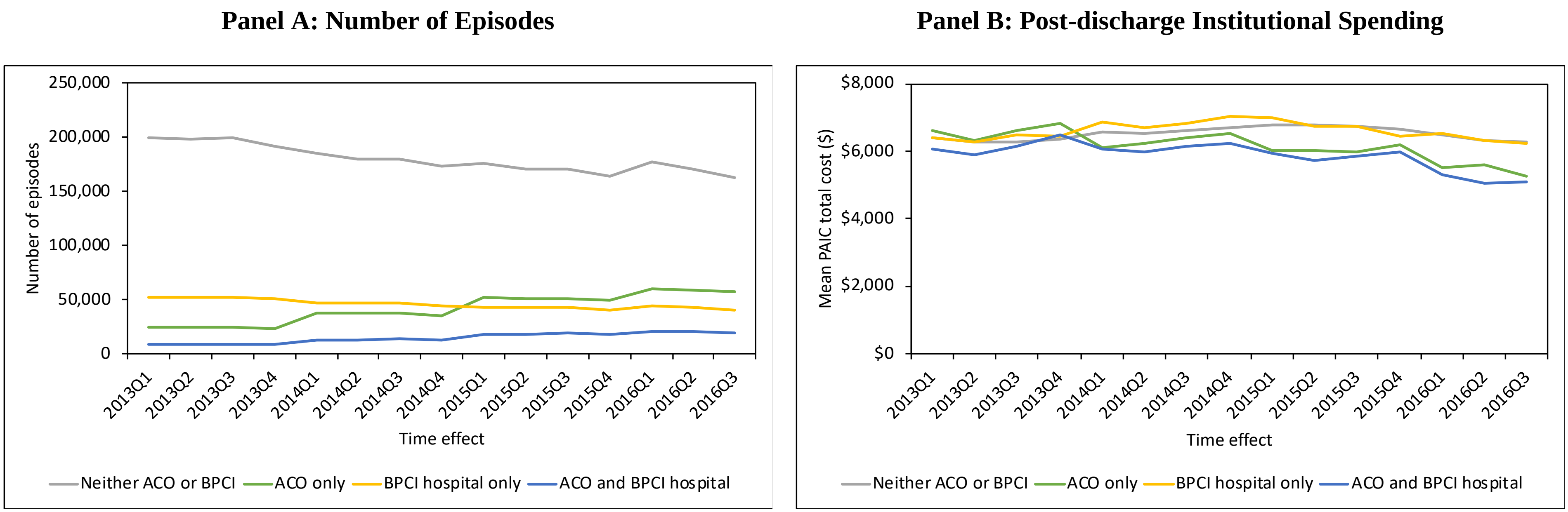

© 2021 Navathe AS et al. *JAMA Health Forum.*

Panel C: Quality and Utilization

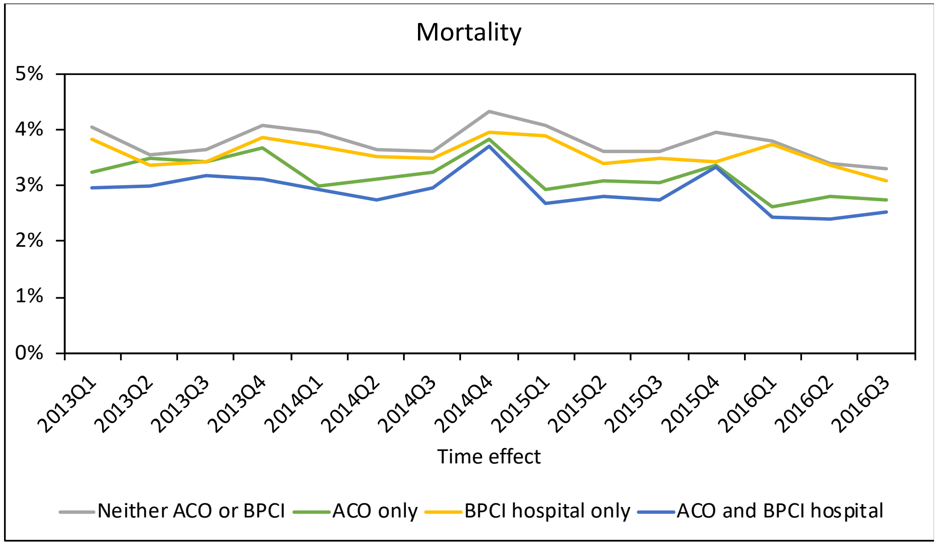

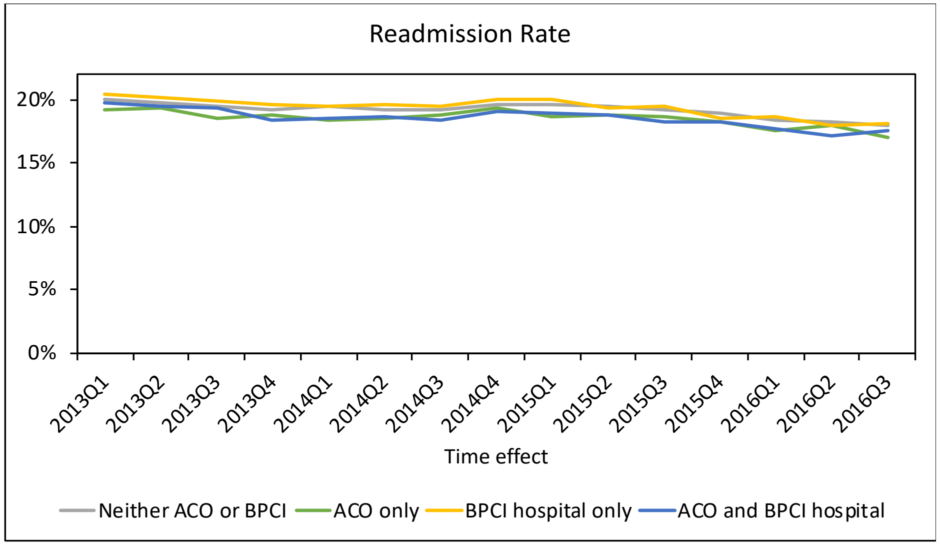

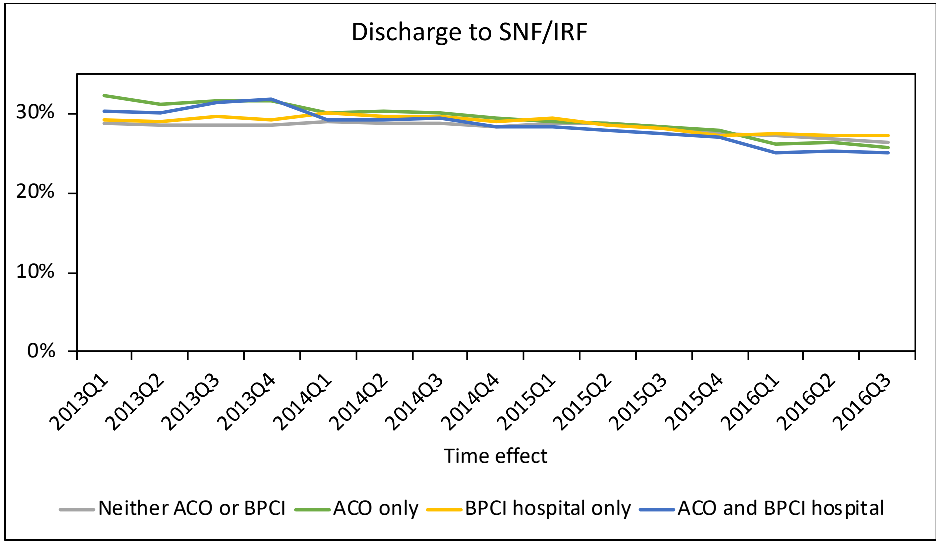

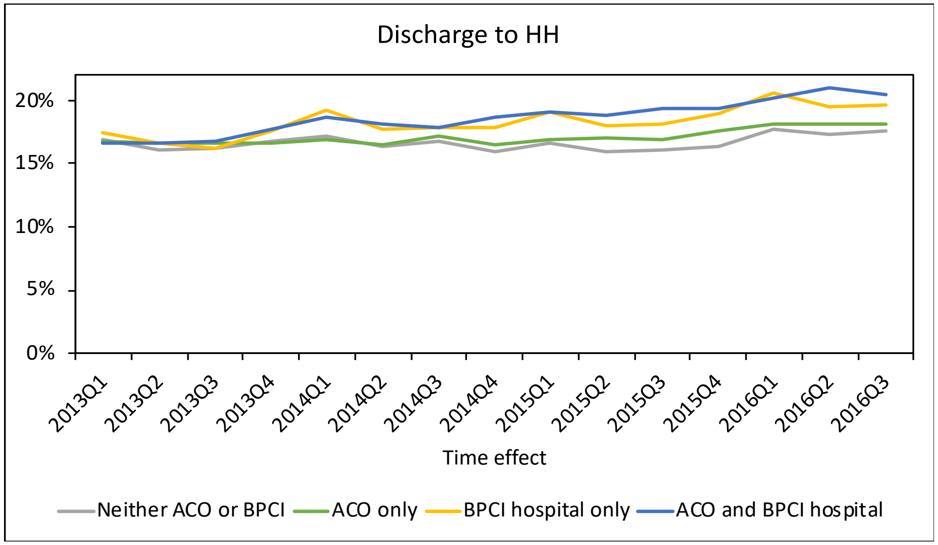

© 2021 Navathe AS et al. *JAMA Health Forum.*

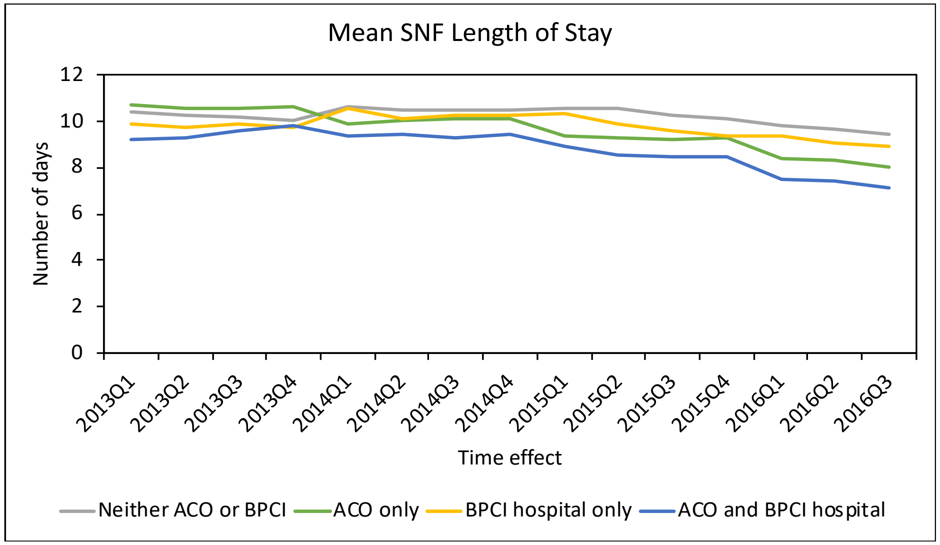

This set of figures shows unadjusted changes in study outcomes for surgical episodes between 2013 Q1 to 2016 Q3 by hospital group: within the ACO group, ACO and BPCI hospitals and ACO only hospitals; and within the Non-ACO group, BPCI only hospitals and Neither ACO or BPCI hospitals. Importantly, these figures do not reflect pre-ACO time periods. **Abbreviations**: ACO, accountable care organization; BPCI, Bundled Payments for Care Improvement; SNF, skilled nursing facility; IRF, inpatient rehabilitation facility; HH, home health agency.

© 2021 Navathe AS et al. *JAMA Health Forum.*

eTable 1. Breakdown of Clinical Episodes by ACO Attribution and Bundled Payment Status

Clinical Episodes	Non-ACO Group		ACO Group	
	**Non-bundled Payment Patients**	**Bundled Payment Patients**	**Non-bundled Payment Patients**	**Bundled Payment Patients**
Sepsis, %	11.5	12.0	11.3	11.7
Simple pneumonia and respiratory infections, %	10.2	9.1	9.5	9.1
Congestive heart failure, %	7.3	7.5	7.3	7.4
Chronic obstructive pulmonary disease, bronchitis, asthma, %	7.5	6.8	7.0	6.5
Stroke, %	6.2	6.7	6.2	6.8
Cardiac arrhythmia, %	5.8	6.1	6.5	6.7
Renal failure, %	5.4	5.6	5.5	5.5
Esophagitis, gastroenteritis, and other digestive disorders, %	5.2	5.1	5.5	5.4
Urinary tract infection, %	5.4	5.3	5.1	5.0
Gastrointestinal hemorrhage, %	4.6	4.8	4.8	5.0
Other respiratory, %	4.0	3.9	3.9	3.8
Cellulitis, %	3.5	3.5	3.4	3.4
Medical non-infectious orthopedic, %	3.1	3.4	3.5	3.5
Nutritional and metabolic disorders, %	3.4	3.3	3.3	3.1
Acute myocardial infarction, %	3.1	3.1	3.2	3.1
Gastrointestinal obstruction, %	2.4	2.3	2.4	2.4
Syncope and collapse, %	2.1	2.3	2.2	2.2
Red blood cell disorders, %	1.8	1.8	1.7	1.7
Transient ischemia, %	1.6	1.8	1.7	1.7
Diabetes, %	1.6	1.5	1.5	1.4
Medical peripheral vascular disorders, %	1.5	1.5	1.5	1.5
Chest pain, %	1.5	1.4	1.4	1.3
Fractures of the femur and hip or pelvis, %	1.1	1.0	1.0	1.0
Atherosclerosis, %	0.5	0.4	0.5	0.4

Abbreviation: ACO, accountable care organization

© 2021 Navathe AS et al. *JAMA Health Forum.*

eTable 2. Characteristics of Patients Admitted for Medical Episodes by Study Period, ACO Attribution, and Bundled Payment Status, 2013-2016

	All patients (n = 7,108,146)							
	Non-ACO Group (n = 5,860,938)				ACO Group (n=1,352,534)			
	Non-bundled Payment Patients		Bundled Payment Patients		Non-bundled Payment Patients		Bundled Payment Patients	
	Pre-bundled Payments (n = 1,497,728)	Post-bundled Payments (n = 4,056,598)	Pre-bundled Payments (n = 260,142)	Post-bundled Payments (n = 690,128)	Pre-bundled Payments (n = 188,423)	Post-bundled Payments (n = 1,000,213)	Pre-bundled Payments (n = 32,091)	Post-bundled Payments (n = 200,978)
**Age, Mean Years (SD)**	77.2 (12.3)	76.7 (12.4)	77.6 (12.3)	77.1 (12.3)	77.9 (11.9)	77.2 (11.8)	78.1 (11.7)	77.6 (11.5)
**Race**								
Black**, %**	10.9	10.5	11.7	11.4	9.6	9.2	8.9	8.8
Hispanic**, %**	2.0	1.8	2.1	2.1	1.8	1.7	1.8	1.4
**Female, %**	59.0	57.6	59.0	57.6	59.6	58.1	59.8	58.0
**Male, %**	41.0	42.4	41.0	42.4	40.4	41.9	40.2	42.0
**Dual-Eligible, %**	26.9	20.8	24.1	19.0	23.7	15.8	21.8	13.7
**Residence in ZIP Code**								
Low-Income**, %**	26.0	25.8	19.9	20.6	19.8	19.5	18.2	15.3
Low-Education**, %**	19.3	18.9	16.6	16.9	14.8	14.2	14.3	12.5
**Elixhauser Comorbidity Index, Mean (SD)**[*]	11.4 (11.4)[a]	10.5 (11.0)[b]	11.6 (11.5)[c]	10.7 (11.1)[d]	11.3 (11.4)[e]	10.1 (10.9)[f]	11.4 (11.5)[g]	10.1 (10.9)[h]
**Most Common Clinical Episodes**								
Simple pneumonia and respiratory infections, %	10.9	9.9	9.9	8.8	10.5	9.3	10.2	9.0
Sepsis, %	9.5	12.2	10.0	12.7	9.0	11.7	9.9	12.0
Chronic obstructive pulmonary disease, bronchitis, asthma, %	8.8	7.1	8.0	6.3	8.5	6.7	7.7	6.3
Congestive heart failure, %	7.5	7.2	7.6	7.4	7.6	7.3	7.8	7.4
Cardiac arrhythmia, %	6.0	5.8	6.3	6.0	6.4	6.5	6.5	6.8
Stroke, %	5.6	6.4	6.0	7.0	5.3	6.4	5.8	7.0
Urinary tract infection, %	5.6	5.3	5.5	5.3	5.6	5.1	5.5	5.0

© 2021 Navathe AS et al. *JAMA Health Forum.*

Renal failure, %	5.3	5.5	5.4	5.6	5.3	5.5	5.3	5.5
Esophagitis, gastroenteritis, and other digestive disorders, %	5.1	5.2	5.1	5.1	5.4	5.6	5.3	5.4
Gastrointestinal hemorrhage, %	4.5	4.7	4.7	4.9	4.7	4.9	4.8	5.0
**Most Common Comorbidities**								
Hypertension, %	80.3	79.5	80.5	80.0	81.6	80.7	81.7	80.9
Fluid and electrolyte disorders, %	45.4	43.6	46.0	44.5	44.8	42.0	45.1	42.3
Chronic lung disease, %	37.3	33.8	35.2	31.9	36.4	32.6	35.5	31.5
Diabetes, %	31.9	28.4	31.3	28.0	31.5	27.1	31.0	26.5
Congestive heart failure, %	30.9	27.6	30.5	27.5	30.9	26.8	31.0	26.8

Abbreviation: ACO, accountable care organization. [*]We reported mean and standard deviation because Elixhauser comorbidity index is a variable included in regression models. Elixhauser comorbidity index is a score that varies from -19 to 89, with larger values corresponding to higher mortality risk. Due to skewness, we also report Median (Interquartile Range):[a,e,g]10.0 (2.0, 18.0), [b,d]10.0 (2.0, 17.0), [c]10.0 (2.0, 19.0), [f,h]9.0 (2.0, 17.0)

© 2021 Navathe AS et al. *JAMA Health Forum.*

eTable 3. Characteristics of Patients Admitted for Surgical Episodes by ACO Attribution and Bundled Payment Status

**Clinical Episodes**	**Non-ACO Group**		**ACO Group**	
	**Non-bundled Payment Patients**	**Bundled Payment Patients**	**Non-bundled Payment Patients**	**Bundled Payment Patients**
Major joint replacement of the lower extremity, %	32.6	31.6	34.0	33.5
Percutaneous coronary intervention, %	9.9	10.6	9.8	10.4
Hip & femur procedures except major joint, %	9.4	8.1	8.3	7.1
Major bowel procedure, %	6.7	6.2	6.8	6.3
Spinal fusion (non-cervical), %	5.2	5.2	5.3	5.3
Cardiac valve, %	3.7	5.1	4.1	5.6
Pacemaker, %	4.0	4.0	4.2	4.1
Coronary artery bypass graft, %	3.4	3.5	3.3	3.7
Other vascular surgery, %	3.5	3.6	3.2	3.3
Major joint replacement of the upper extremity, %	3.2	3.0	3.4	3.2
Cervical spinal fusion, %	3.0	2.9	2.8	2.7
Major cardiovascular procedure, %	2.7	3.0	2.7	2.9
Back & neck except spinal fusion, %	2.5	2.5	2.4	2.3
Lower extremity and humerus procedure except hip, foot, femur, %	2.7	2.3	2.4	2.1
Revision of the hip or knee, %	2.1	2.4	2.0	2.2
Amputation, %	1.5	1.4	1.2	1.1
Cardiac defibrillator, %	1.0	1.2	0.9	1.1
Double joint replacement of the lower extremity, %	0.6	0.7	0.7	0.7
Combined anterior posterior spinal fusion, %	0.7	0.7	0.7	0.5
Other knee procedures, %	0.6	0.6	0.5	0.5
Pacemaker device replacement or revision, %	0.4	0.5	0.4	0.4
Complex non-cervical spinal fusion, %	0.4	0.5	0.4	0.4
Removal of orthopedic devices, %	0.5	0.5	0.4	0.4
AICD generator or lead, %	0.1	0.2	0.1	0.2

Abbreviations: ACO, accountable care organization. AICD, automatic implantable cardioverter defibrillator.

© 2021 Navathe AS et al. *JAMA Health Forum.*

eTable 4. Characteristics of Patients Admitted for Surgical Episodes by Study Period, ACO Attribution, and Bundled Payment Status, 2013-2016

	All patients (n = 3,675,962)							
	Non-ACO Group (n = 2,967,790)				ACO Group (n = 732,458)			
	Non-bundled Payment Patients		Bundled Payment Patients		Non-bundled Payment Patients		Bundled Payment Patients	
	Pre-bundled Payments (n = 585,043)	Post-bundled Payments (n = 1,878,815)	Pre-bundled Payments (n = 153,647)	Post-bundled Payments (n = 473,004)	Pre-bundled Payments (n = 70,208)	Post-bundled Payments (n = 482,627)	Pre-bundled Payments (n = 24,884)	Post-bundled Payments (n = 168,449)
**Age, Mean Years (SD)**	74.8 (10.1)	74.6 (10.2)	74.8 (10.0)	74.6 (10.1)	75.2 (9.8)	74.9 (9.7)	75.0 (9.7)	74.9 (9.4)
**Race**								
Black, %	6.2	6.1	7.3	7.2	5.8	5.7	5.8	6.2
Hispanic, %	1.2	1.2	1.2	1.2	1.1	1.0	0.9	0.9
**Female, %**	57.0	56.5	56.4	55.9	57.6	56.6	56.8	56.0
**Male, %**	43.0	43.6	43.6	44.1	42.4	43.4	43.2	44.0
**Dual-Eligible, %**	26.9	20.8	24.1	19.0	23.7	15.8	21.8	13.7
**Residence in ZIP Code**								
Low-Income, %	22.2	22.2	18.8	18.8	16.7	16.7	15.8	13.6
Low-Education, %	15.2	15.2	14.2	14.1	11.2	11.2	11.4	10.6
**Elixhauser Comorbidity Index, Mean (SD)***	4.1 (9.1)[a]	3.9 (9.1)[b]	4.2 (9.3)[c]	4.0 (9.2)[d]	4.0 (9.2)[e]	3.7 (8.9)[f]	4.0 (9.1)[g]	3.8 (9.1)[h]
**Most Common Clinical Episodes**								
Major joint replacement of the lower extremity, %	31.5	32.9	30.4	31.9	32.6	34.2	31.8	33.7
Percutaneous coronary intervention, %	10.7	9.6	11.7	10.2	10.7	9.7	11.7	10.2
Hip & femur procedures except major joint, %	8.8	9.5	7.4	8.3	8.2	8.3	7.2	7.1
Major bowel procedure, %	6.7	6.6	6.2	6.2	6.9	6.8	6.2	6.4
Spinal fusion (non-cervical), %	5.0	5.2	5.1	5.3	4.8	5.3	4.9	5.3
Pacemaker, %	4.2	3.9	4.3	3.9	4.5	4.1	4.2	4.0
Other vascular surgery, %	4.0	3.3	4.1	3.4	3.9	3.2	3.7	3.2

© 2021 Navathe AS et al. *JAMA Health Forum.*

Cardiac valve, %	3.3	3.8	4.7	5.3	3.3	4.2	4.8	5.7
Coronary artery bypass graft, %	3.2	3.4	3.4	3.5	2.9	3.4	3.5	3.7
Major joint replacement of the upper extremity, %	2.8	3.3	2.7	3.1	3.0	3.4	2.8	3.2
**Most Common Comorbidities**								
Hypertension, %	77.0	76.3	77.3	76.8	78.0	77.3	77.6	77.8
Diabetes, %	25.2	23.4	25.3	23.1	25.0	22.7	24.1	22.5
Chronic lung disease, %	21.6	21.4	21.4	21.1	21.1	20.9	21.4	20.7
Hypothyroidism, %	19.2	19.4	18.9	19.3	19.3	19.9	18.9	19.3
Obesity, %	15.0	15.7	16.0	17.5	15.3	16.5	15.3	17.6

Abbreviation: ACO, accountable care organization. *We reported mean and standard deviation because Elixhauser comorbidity index is a variable included in regression models. Elixhauser comorbidity index is a score that varies from -19 to 89, with larger values corresponding to higher mortality risk. Due to skewness, we also report Median (Interquartile Range): [a-h]0.0 (-1.0, 8.0)

© 2021 Navathe AS et al. *JAMA Health Forum.*

eTable 5. ACO Characteristics, 2013-2016

	2013	2014	2015	2016
**No. of ACOs**	220	333	392	432
**No. of Attributed Beneficiaries, Mean**	15,429.9	16,005.5	18,546.5	18,250.1
**ACO includes a Hospital, n (%)**	34 (15.5%)	132 (39.6%)	193 (49.2%)	232 (53.7%)
**Median No. of Hospitals per ACO (if ACO includes a Hospital) (Min, Max)**	4 (1, 11)	2 (1, 15)	2 (1, 30)	3 (1, 31)
**Median No. of Physicians per ACO (Min, Max)**	227 (14, 2,403)	207 (5, 5,684)	286 (18, 6,208)	305 (16, 5,357)
**Median No. of Post-Acute Care Facilities per ACO (Min, Max)**	2 (1, 26)	2 (1, 40)	2 (1, 116)	2 (1, 173)

Abbreviation: ACO, accountable care organization.

© 2021 Navathe AS et al. *JAMA Health Forum.*

eTable 6. Hospital Characteristics by Bundled Payment Status, 2011-2013

	Medical Episodes		Surgical Episodes	
	**Non-bundled Payment Hospitals**	**Bundled Payment Hospitals**	**Non-bundled Payment Hospitals**	**Bundled Payment Hospitals**
**Hospitals, No.**	2,893	293	2,697	365
**Total Episodes, No.**	1,826,024	316,562	669,770	182,649
**Urban Status, %**	69.4	91.8	71.6	93.7
**Total Hospital Beds**				
Small (<100), %	34.3	7.8	32.7	9.0
Medium (100-399), %	53.0	65.2	54.9	61.1
Large (≥400), %	12.7	27.0	12.4	29.9
**Ownership**				
For Profit, %	22.1	21.8	24.4	19.5
Government, %	16.8	4.8	14.8	4.4
Not-for-Profit, %	61.1	73.4	60.8	76.2
**Teaching Hospital**				
Major Teaching, %	23.2	45.4	23.3	47.1
Minor Teaching, %	10.9	10.9	11.5	9.9
Non-Teaching, %	65.1	43.3	64.6	42.5

This table used American Hospital Data as the primary source of data and the Medicare Provider of Service file as a secondary source to address missingness.

© 2021 Navathe AS et al. *JAMA Health Forum.*

eTable 7. Unadjusted Changes in Medical Episode Outcomes Prebundled and Postbundled Payments, by ACO Attribution

	**Non-ACO Group**				**ACO Group**			
	**Non-bundled Payment Patients**		**Bundled Payment Patients**		**Non-bundled Payment Patients**		**Bundled Payment Patients**	
	**Pre-bundled Payments (n = 1,621,781)**	**Post-bundled Payments (n = 5,614,220)**	**Pre-bundled Payments (n = 281,832)**	**Post-bundled Payments (n = 952,167)**	**Pre-bundled Payments (n = 204,243)**	**Post-bundled Payments (n = 1,442,783)**	**Pre-bundled Payments (n = 34,730)**	**Post-bundled Payments (n = 291,705)**
**Post-discharge Institutional Spending, Mean $**	$6,135	$6,634	$6,571	$7,065	$6,328	$6,225	$6,479	$6,143
**Mortality Rate, %**	12.5	13.0	12.6	13.2	10.9	11.1	11.1	11.1
**Readmission Rate, %**	27.6	28.6	27.7	28.9	27.3	28.1	27.5	27.9
**Discharge to SNF/IRF, %**	18.8	19.5	20.0	20.6	19.8	19.3	19.4	18.7
**Discharge to HH, %**	7.9	7.1	8.7	7.7	8.5	7.7	10.4	8.8
**SNF/IRF Length of Stay, Mean Days**	9.4	9.7	9.7	9.8	9.5	8.8	9.3	8.5

Abbreviations: ACO, accountable care organization; SNF, skilled nursing facility; IRF, inpatient rehabilitation facility; HH, home health agency. This table shows unadjusted changes in outcomes for medical episodes, before versus after the start of BPCI. These before-after comparisons are displayed separately for four different patient groups: non-bundled payment patients in the Non-ACO group; bundled payment patients in the Non-ACO group; non-bundled payment patients in the ACO group; and bundled payment patients in the ACO group. Values in this table denote changes in outcomes prior to multivariable analysis.

© 2021 Navathe AS et al. *JAMA Health Forum.*

eTable 8. Percent Changes in Medical and Surgical Episode Outcomes Associated With Bundled Payments Among Non-ACO and ACO-Attributed Patients, 2013 Quarter 1 to 2016 Quarter 3

	**Medical Episodes**			**Surgical Episodes**		
	**ACO Group**	**Non-ACO Group**	**ACO versus Non-ACO difference**	**ACO Group**	**Non-ACO Group**	**ACO versus Non-ACO difference**
	**% Change**	**% Change**	**Percentage Point Change**	**% Change**	**% Change**	**Percentage Point Change**
**Post-discharge Institutional Spending**	-8.23%	-3.14%	-5.08 p.p.	-12.91%	-10.96%	-1.95 p.p.
**Mortality Rate**	-1.60%	0.02%	-1.61 p.p.	0.35%	-0.22%	0.57 p.p.
**Readmission Rate**	-4.07%	-0.51%	-3.56 p.p.	-7.69%	-3.48%	-4.22 p.p.
**Discharge to SNF/IRF**	-2.27%	0.76%	-3.04 p.p.	-9.70%	-9.82%	0.12 p.p.
**Discharge to HH**	7.62%	4.04%	3.58 p.p.	21.32%	18.88%	2.44 p.p.
**SNF/IRF Length of Stay**	-9.35%	-4.07%	-5.28 p.p.	-11.83%	-11.02%	-0.81 p.p.

This table shows the changes in episode outcomes by ACO attribution in percent or percentage points terms. **Abbreviations**: ACO, accountable care organization; SNF, skilled nursing facility; IRF, inpatient rehabilitation facility; HH, home health agency.

© 2021 Navathe AS et al. *JAMA Health Forum.*

eFigure 5. Changes in Medical Episode SNF Length of Stay Associated With Bundled Payments by ACO Attribution, 2013-2016

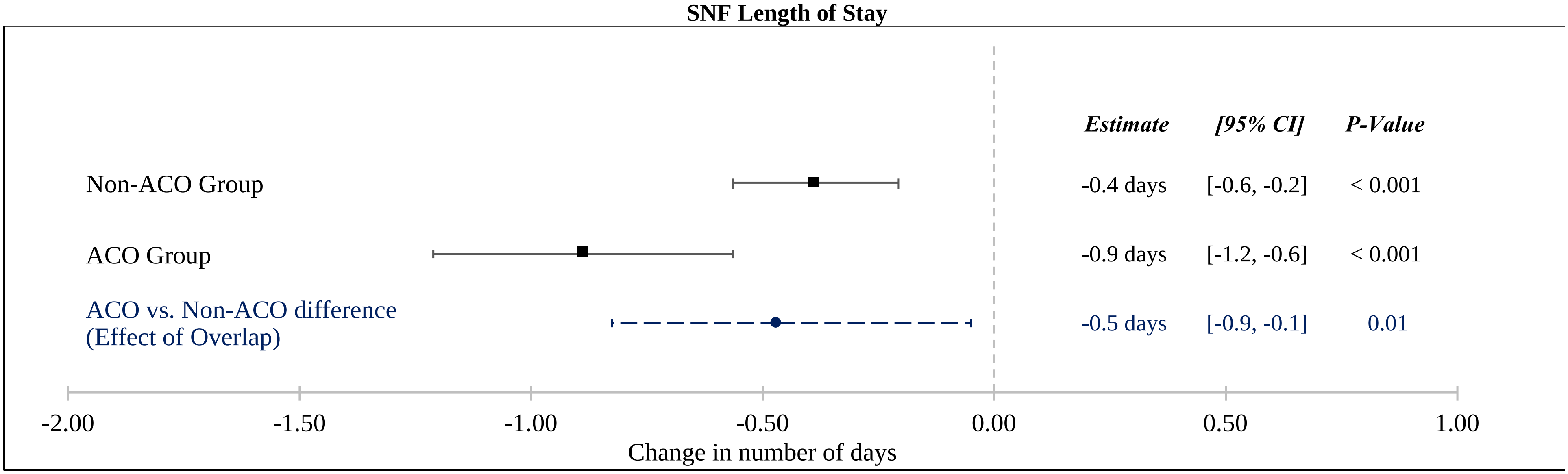

Abbreviations: ACO, accountable care organization; SNF, skilled nursing facility This figure shows adjusted changes in SNF length of stay for medical episodes among bundled payments versus non-bundled payments patients, separately in the Non-ACO group and in the ACO group. This figure also shows "ACO vs. Non-ACO difference" – the adjusted changes in SNF length of stay for bundled payment patients in the ACO vs. Non-ACO group. Point estimates and confidence intervals to the left of the dotted line at 0 denote differentially lower SNF length of stay, while point estimates and confidence intervals to the right of the dotted line at 0 denote differentially higher SNF length of stay.

© 2021 Navathe AS et al. *JAMA Health Forum.*

eTable 9. Unadjusted Changes in Surgical Episode Outcomes Prebundled and Postbundled Payments, by ACO Attribution

	Non-ACO Group				ACO Group			
	Non-bundled Payment Patients		Bundled Payment Patients		Non-bundled Payment Patients		Bundled Payment Patients	
	Pre-bundled Payments (n = 598,010)	Post-bundled Payments (n = 2,101,288)	Pre-bundled Payments (n = 157,179)	Post-bundled Payments (n = 529,221)	Pre-bundled Payments (n = 71,760)	Post-bundled Payments (n = 550,497)	Pre-bundled Payments (n = 25,470)	Post-bundled Payments (n = 192,410)
**Post-discharge Institutional Spending, Mean $**	$6,317	$6,567	$6,376	$6,655	$6,509	$5,958	$6,034	$5,736
**Mortality Rate, %**	3.8	3.8	3.5	3.6	3.4	3.1	3.0	2.8
**Readmission Rate, %**	19.8	19.1	20.2	19.2	19.0	18.3	19.5	18.3
**Discharge to SNF/IRF, %**	28.5	28.1	29.2	28.6	31.7	28.2	30.5	27.4
**Discharge to HH, %**	16.4	16.7	16.8	18.9	16.7	17.2	16.7	19.3
**SNF/IRF Length of Stay, Mean Days**	10.3	10.2	9.9	9.8	10.6	9.2	9.4	8.5

Abbreviations: ACO, accountable care organization; SNF, skilled nursing facility; IRF, inpatient rehabilitation facility; HH, home health agency. This table shows unadjusted changes in outcomes for surgical episodes, before versus after the start of BPCI. These before-after comparisons are displayed separately for four different patient groups: non-bundled payment patients in the non-ACO group; bundled payment patients in the non-ACO group; non-bundled payment patients in the ACO group; and bundled payment patients in the ACO group. Values in this table denote changes in outcomes prior to multivariable analysis.

© 2021 Navathe AS et al. *JAMA Health Forum.*

eFigure 6. Changes in Surgical Episode SNF Length of Stay Associated With Bundled Payments by ACO Attribution, 2013-2016

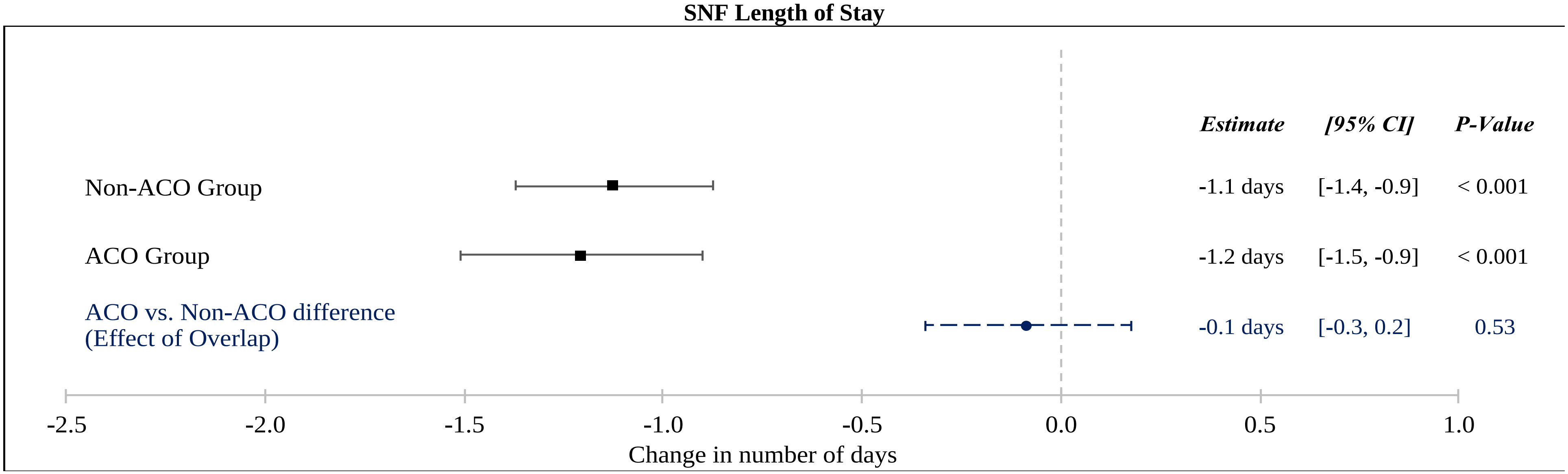

Abbreviations: ACO, accountable care organization; SNF, skilled nursing facility. This figure shows adjusted changes in SNF length of stay for surgical episodes among bundled payments versus non-bundled payments patients, separately in the Non-ACO group and in the ACO group. This figure also shows "ACO vs. Non-ACO difference" – the adjusted changes in SNF length of stay for bundled payment patients in the ACO vs. Non-ACO group. Point estimates and confidence intervals to the left of the dotted line at 0 denote differentially lower SNF length of stay, while point estimates and confidence intervals to the right of the dotted line at 0 denote differentially higher SNF length of stay.

© 2021 Navathe AS et al. *JAMA Health Forum.*

eFigure 7. Sensitivity Analysis for Medical Episodes, Using Models Without ACO Fixed Effects, 2013-2016

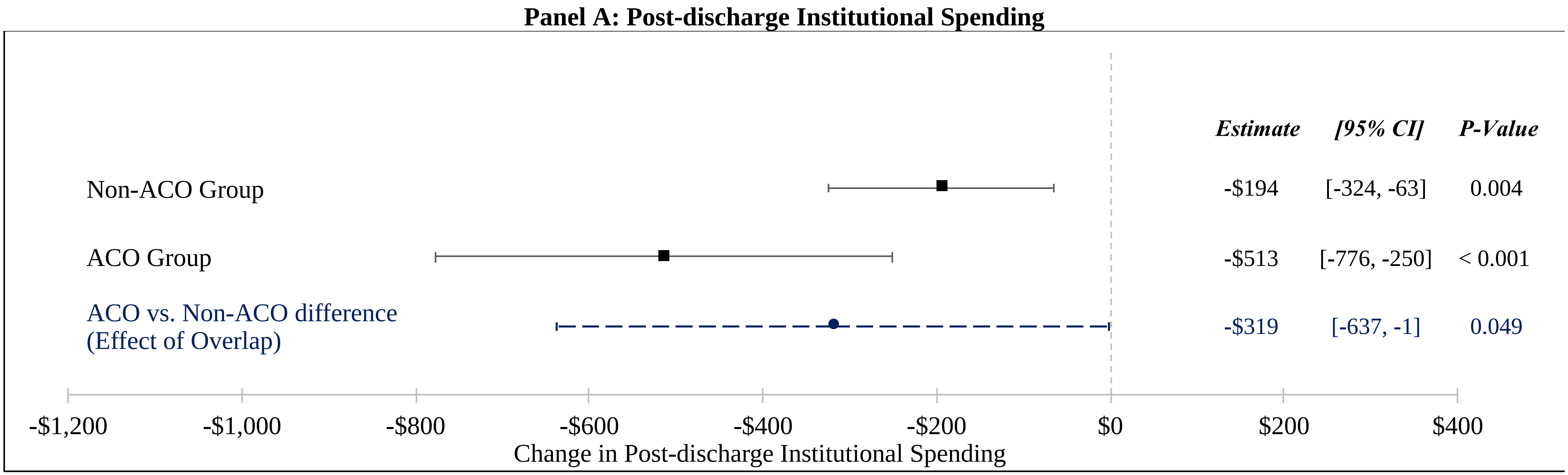

© 2021 Navathe AS et al. *JAMA Health Forum.*

Panel B: Secondary Outcomes

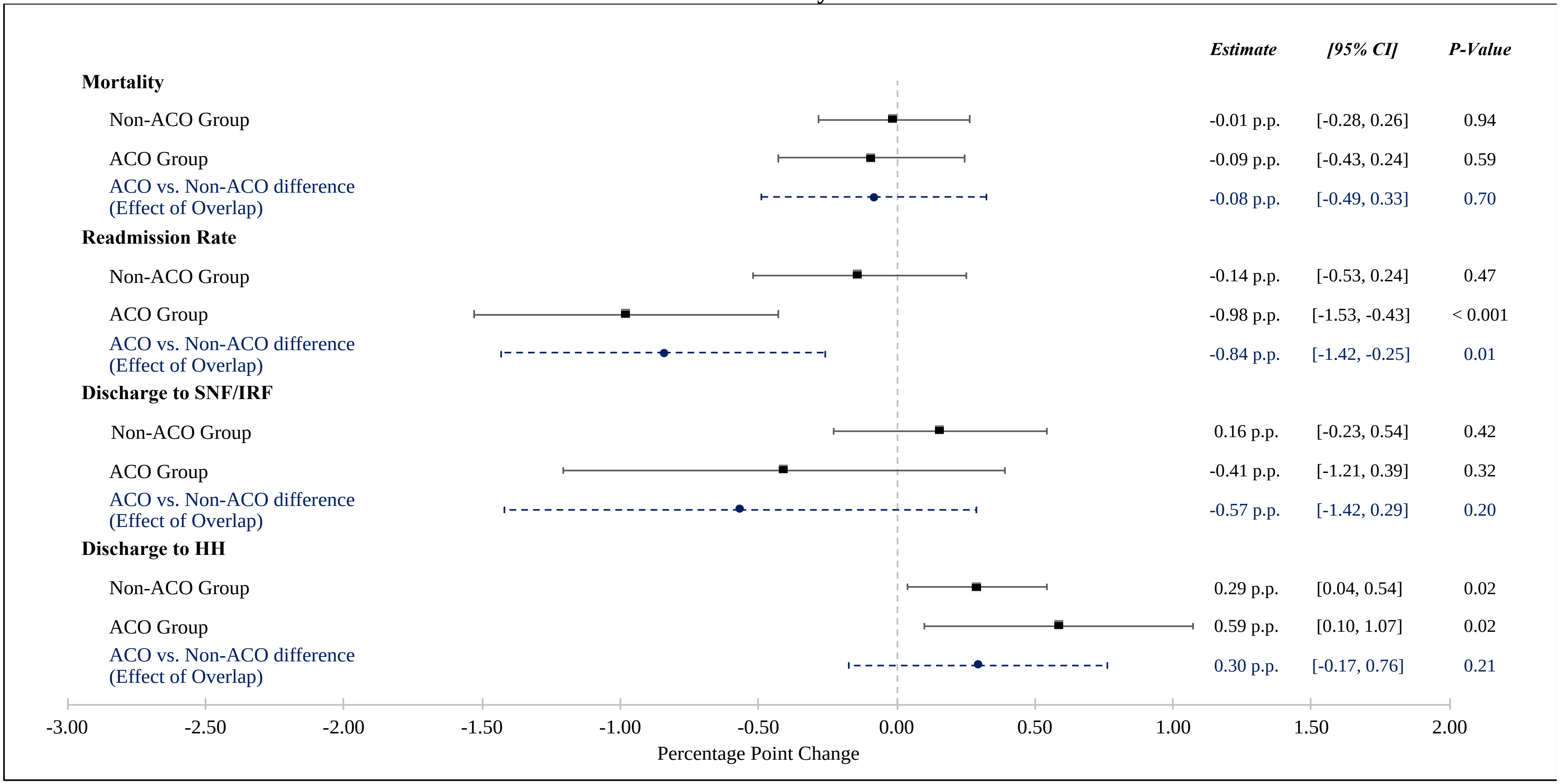

© 2021 Navathe AS et al. *JAMA Health Forum.*

Panel C: SNF Length of Stay

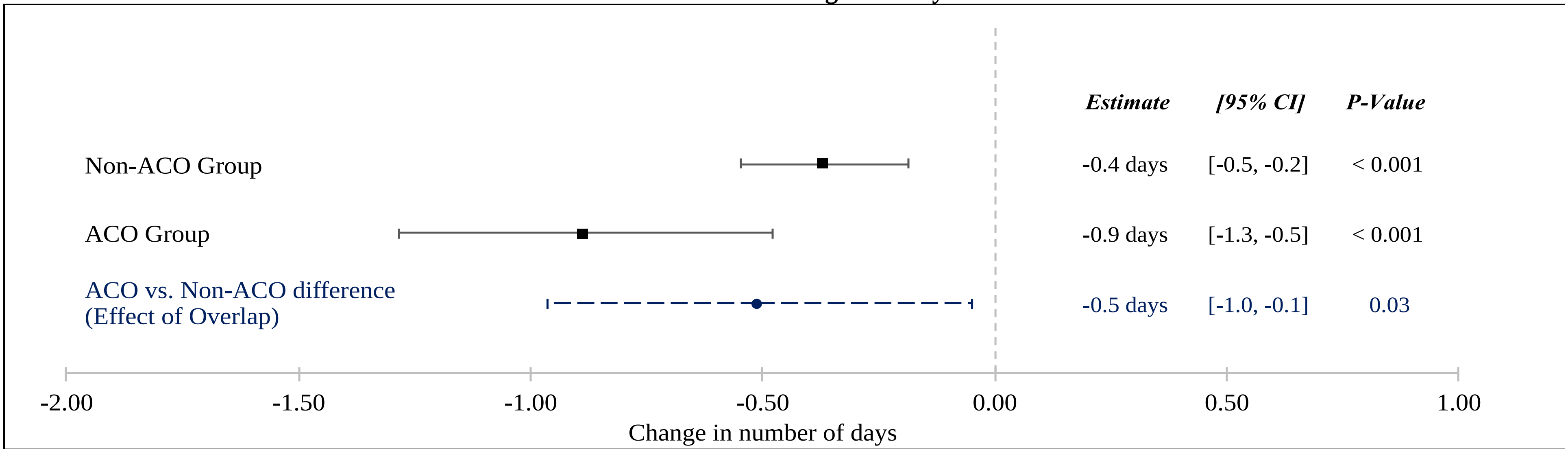

Abbreviations: ACO, accountable care organization; SNF, skilled nursing facility; IRF, inpatient rehabilitation facility; HH, home health agency. These figures shows adjusted changes in post-discharge institutional spending, secondary outcomes, and SNF length of stay for medical episodes among bundled payments versus non-bundled payments patients, separately in the Non-ACO group and in the ACO group. This figure also shows “ACO vs. Non-ACO difference” – the adjusted changes in outcomes for bundled payment patients in the ACO vs. Non-ACO group. Point estimates and confidence intervals to the left of the dotted line at 0 denote differentially lower outcomes (e.g., shorter SNF length of stay), while point estimates and confidence intervals to the right of the dotted line at 0 denote differentially higher outcomes (e.g., longer SNF length of stay).

© 2021 Navathe AS et al. *JAMA Health Forum.*

eFigure 8. Sensitivity Analysis for Surgical Episodes, Using Models Without ACO Fixed Effects, 2013-2016

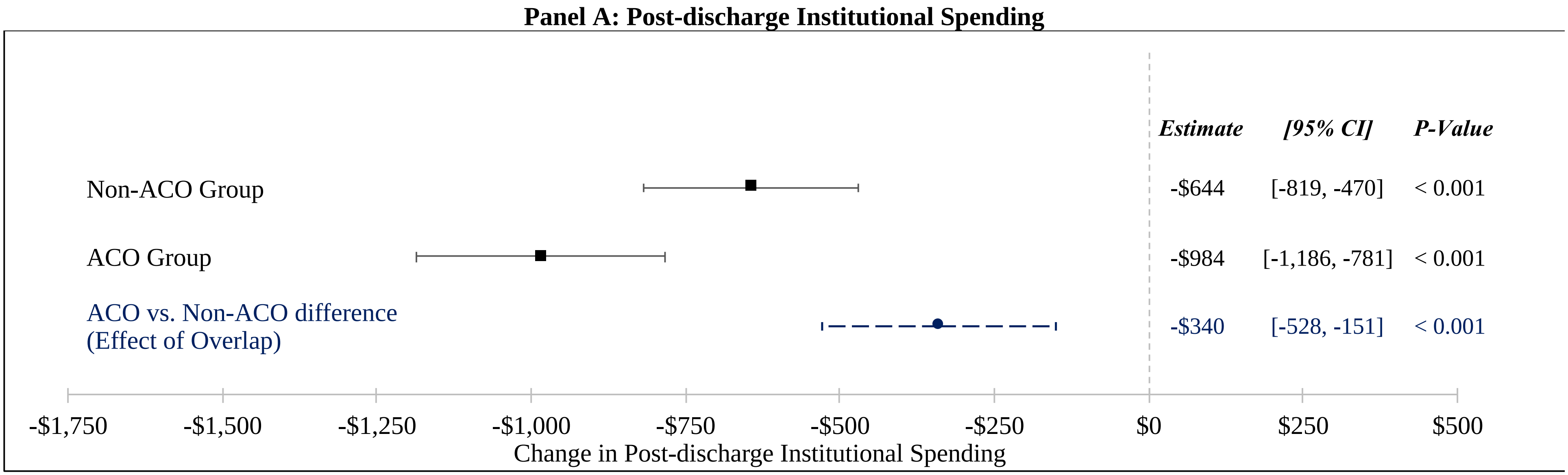

© 2021 Navathe AS et al. *JAMA Health Forum.*

Panel B: Secondary Outcomes

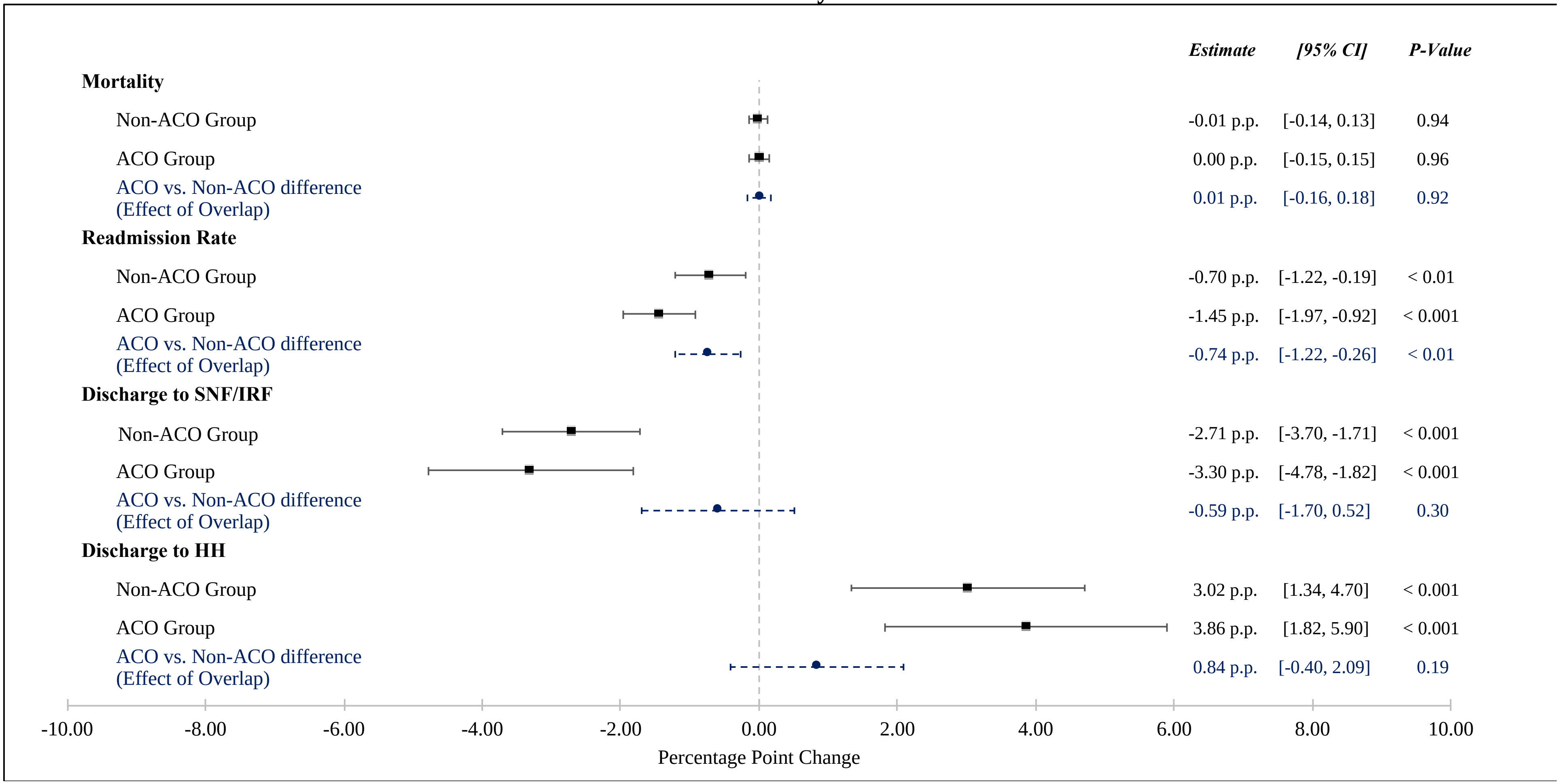

© 2021 Navathe AS et al. *JAMA Health Forum.*

Panel C: SNF Length of Stay

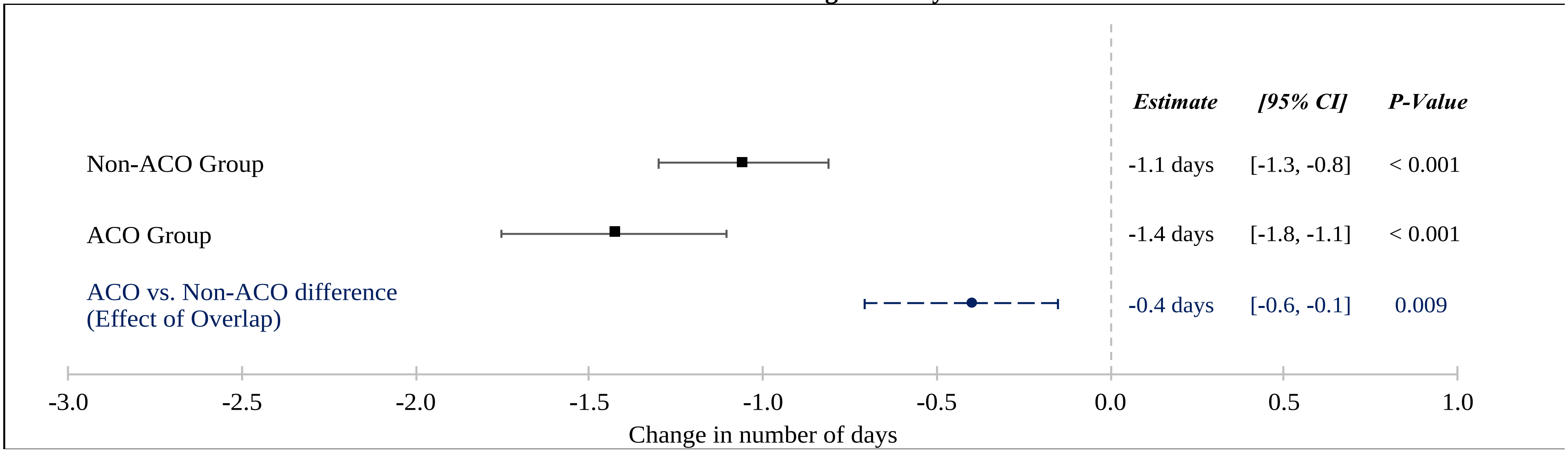

Abbreviations: ACO, accountable care organization; SNF, skilled nursing facility; IRF, inpatient rehabilitation facility; HH, home health agency. These figures shows adjusted changes in Post-discharge institutional spending, secondary outcomes, and SNF length of stay for surgical episodes among bundled payments versus non-bundled payments patients, separately in the Non-ACO group and in the ACO group. This figure also shows “ACO vs. Non-ACO difference” – the adjusted changes in outcomes for bundled payment patients in the ACO vs. Non-ACO group. Point estimates and confidence intervals to the left of the dotted line at 0 denote differentially lower outcomes (e.g., shorter SNF length of stay), while point estimates and confidence intervals to the right of the dotted line at 0 denote differentially higher outcomes (e.g., longer SNF length of stay).

© 2021 Navathe AS et al. *JAMA Health Forum.*

eFigure 9. Sensitivity Analysis for Medical Episodes Controlling for ACO Years of Experience, 2013-2016

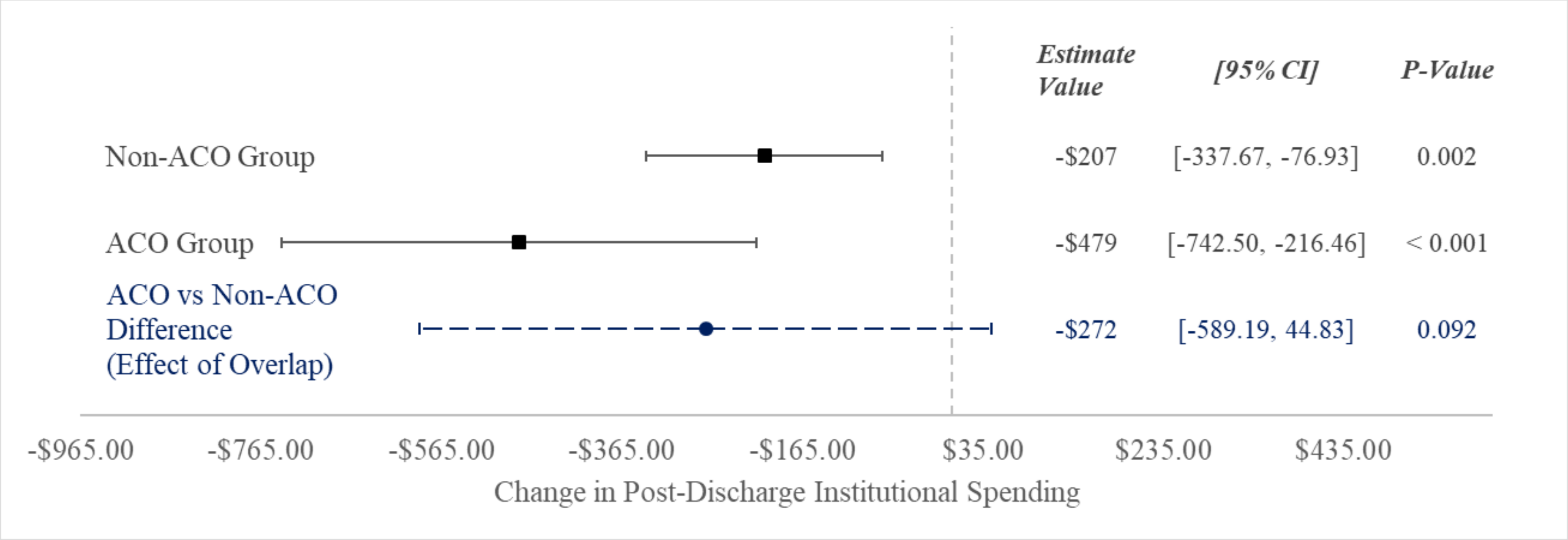

This figure includes additional controls for the number of years of experience an ACO had in the Medicare Shared Savings Program at the time of the episode. The model does not uses the number of years in the ACO and the interaction between the number of years in the ACO and the ACO-attribution indicator variable, without ACO fixed effects (due to collinearity). The results are similar to the main results that attribution to an ACO is associated with decreased episode spending, although the statistical significance decreased moderately. **Abbreviation**: ACO, accountable care organization.

© 2021 Navathe AS et al. *JAMA Health Forum.*

eFigure 10. Sensitivity Analysis for Surgical Episodes Controlling for ACO Years of Experience, 2013-2016

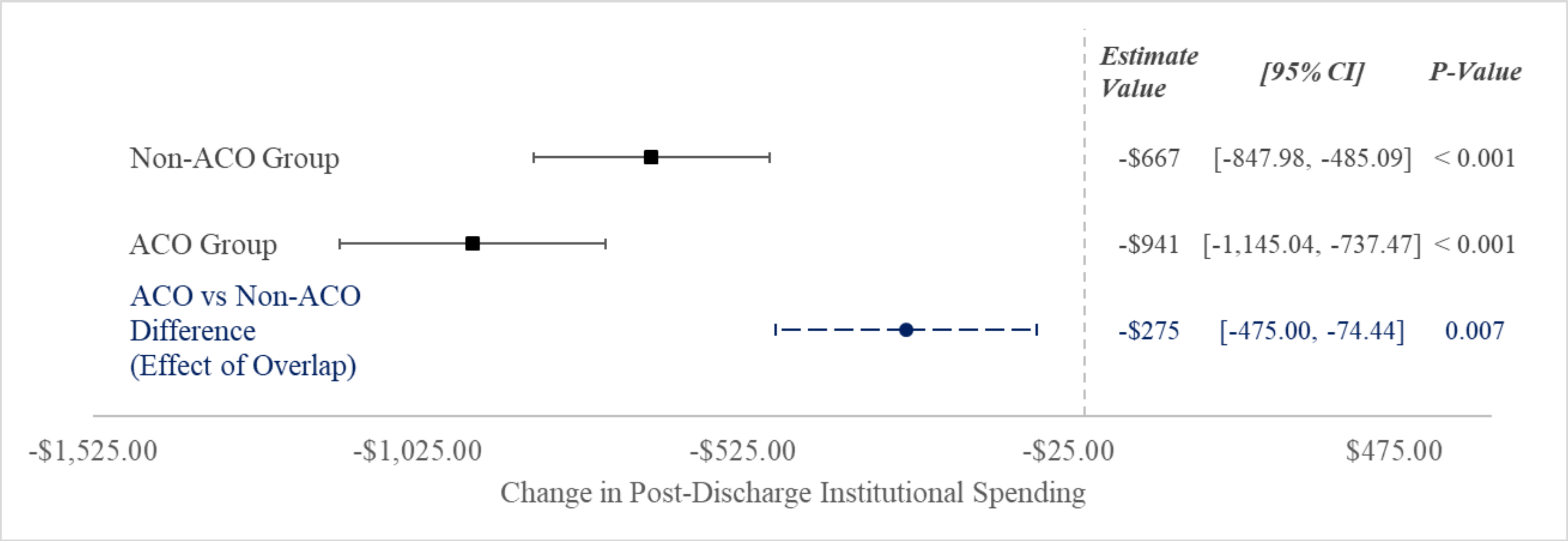

This figure includes additional controls for the number of years of experience an ACO had in the Medicare Shared Savings Program at the time of the episode. The model does not uses the number of years in the ACO and the interaction between the number of years in the ACO and the ACO-attribution indicator variable, without ACO fixed effects (due to collinearity). The results are similar to the main results, although here attribution to an ACO is associated with decreased episode spending that is statitiscally significant. **Abbreviation**: ACO, accountable care organization.

© 2021 Navathe AS et al. *JAMA Health Forum.*

eFigure 11. Sensitivity Analysis for Changes in Postdischarge Institutional Spending in Medical Episodes, Using Generalized Linear Models With Log Link and Gamma Distribution, 2013-2016

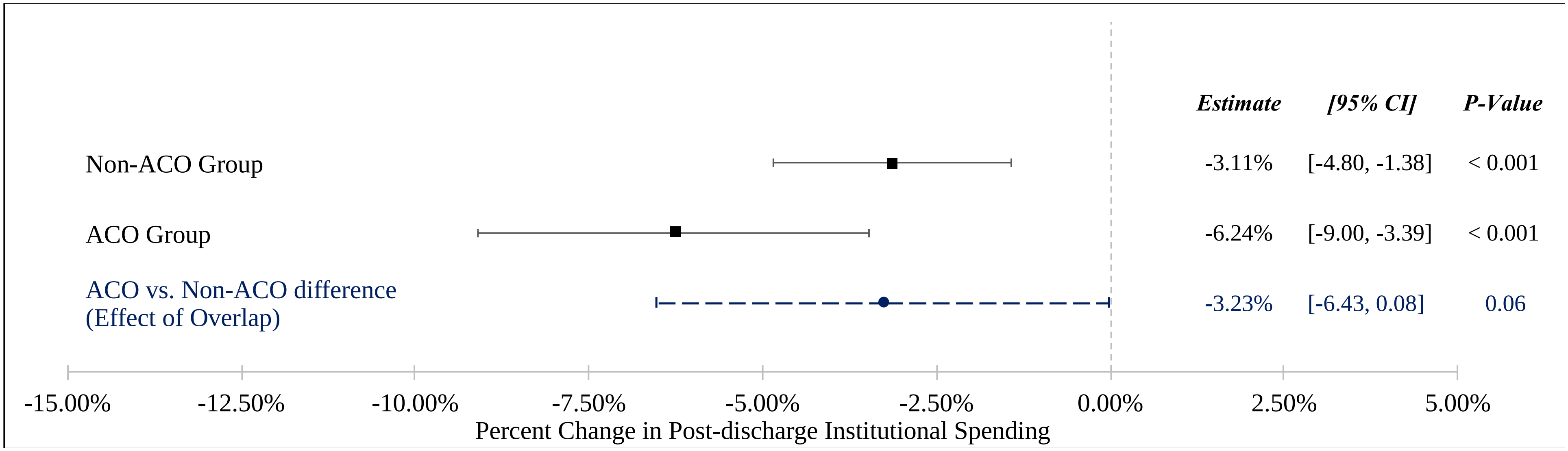

Abbreviation: ACO, accountable care organization. This figure shows adjusted changes in post-discharge institutional spending for medical episodes among bundled payments versus non-bundled payments patients, separately in the Non-ACO group and in the ACO group. This figure also shows "ACO vs. Non-ACO difference" – the adjusted changes in post-discharge institutional spending for bundled payment patients in the ACO vs. Non-ACO group. Point estimates and confidence intervals to the left of the dotted line at 0 denote differentially lower post-discharge institutional spending, while point estimates and confidence intervals to the right of the dotted line at 0 denote differentially higher post-discharge institutional spending.

© 2021 Navathe AS et al. *JAMA Health Forum.*

eFigure 12. Sensitivity Analysis for Changes in Postdischarge Institutional Spending in Surgical Episodes, Using Generalized Linear Models With Log Link and Gamma Distribution, 2013-2016

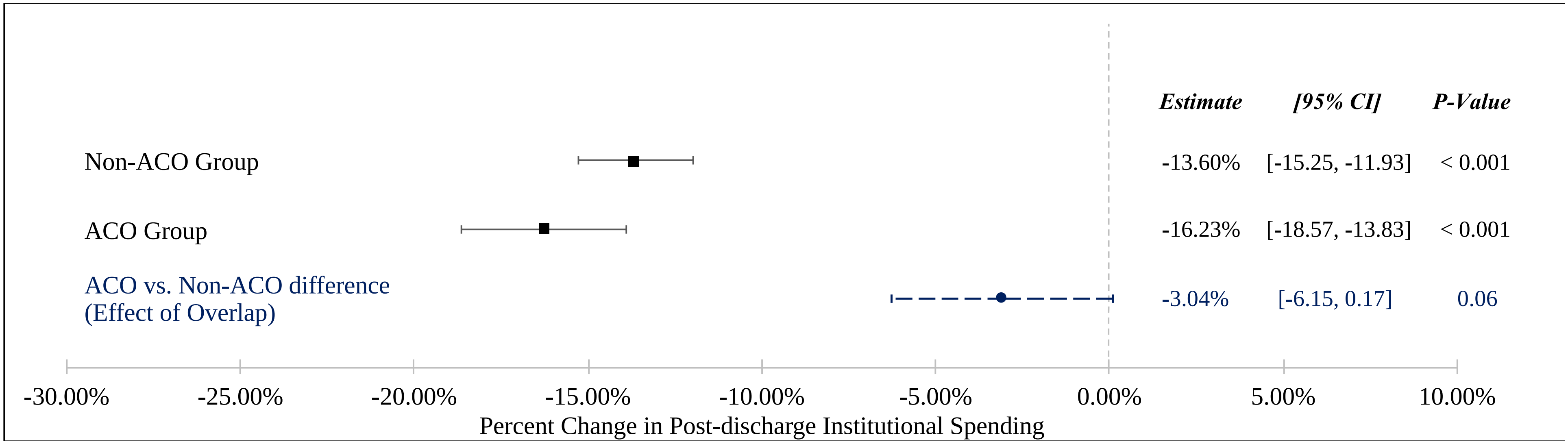

Abbreviation: ACO, accountable care organization. This figure shows adjusted changes in post-discharge institutional spending for surgical episodes among bundled payments versus non-bundled payments patients, separately in the Non-ACO group and in the ACO group. This figure also shows “ACO vs. Non-ACO difference” – the adjusted changes in post-discharge institutional spending for bundled payment patients in the ACO vs. Non-ACO group. Point estimates and confidence intervals to the left of the dotted line at 0 denote differentially lower post-discharge institutional spending, while point estimates and confidence intervals to the right of the dotted line at 0 denote differentially higher post-discharge institutional spending.

© 2021 Navathe AS et al. *JAMA Health Forum.*

eTable 10. Sensitivity Analysis for Medical Episodes, ACOs Including Hospitals, 2013-2016

	Estimate	95% CI	P-Value
**Post-discharge Institutional Spending, Mean $**	$238	[-455, 931]	0.50
**Mortality Rate, p.p.**	-0.54 p.p.	[-1.45,0.38]	0.25
**Readmission Rate, p.p.**	0.72 p.p.	[-0.58, 2.02]	0.28
**Discharge to SNF/IRF, p.p.**	-0.17 p.p.	[-0.19,1.54]	0.85
**Discharge to HH, p.p.**	0.08 p.p.	[-0.87,1.04]	0.17
**SNF/IRF Length of Stay, Mean Days**	0.2 days	[-0.6, 1.1]	0.61

Abbreviations: ACO, accountable care organization; SNF, skilled nursing facility; IRF, inpatient rehabilitation facility; HH, home health agency. This table shows adjusted changes in outcomes for medical episodes among bundled payment patients in the ACO group vs. bundled payment patients in the Non-ACO group. Values with 95% confidence intervals that cross 0 and that have $p>0.05$ suggest no differential changes in outcomes between these two groups of patients.

© 2021 Navathe AS et al. *JAMA Health Forum.*

eTable 11. Sensitivity Analysis for Surgical Episodes, ACOs Including Hospitals, 2013-2016

	Estimate	95% CI	P-Value
**Post-discharge Institutional Spending, Mean $**	$345	[-64,754]	0.10
**Mortality Rate, p.p.**	0.09 p.p.	[-0.29,0.46]	0.64
**Readmission Rate, p.p.**	-0.23 p.p.	-1.18,0.71]	0.63
**Discharge to SNF/IRF, p.p.**	2.62 p.p.	[0.48,4.8]	0.02
**Discharge to HH, p.p.**	-0.36 p.p.	[-2.96,2.23]	0.78
**SNF/IRF Length of Stay, Mean Days**	0.63 days	[0.08,1.17]	0.025

Abbreviations: ACO, accountable care organization; SNF, skilled nursing facility; IRF, inpatient rehabilitation facility; HH, home health agency. This table shows adjusted changes in outcomes for surgical episodes among bundled payment patients in the ACO group vs. bundled payment patients in the Non-ACO group. Values with 95% confidence intervals that cross 0 and that have p>0.05 suggest no differential changes in outcomes between these two groups of patients.

© 2021 Navathe AS et al. *JAMA Health Forum.*
